# Narrative theory and the dynamics of popular movies

**DOI:** 10.3758/s13423-016-1051-4

**Published:** 2016-05-03

**Authors:** James E. Cutting

**Affiliations:** Department of Psychology, Uris Hall, Cornell University, 109 Tower Road, Ithaca, NY 14853-7601 USA

**Keywords:** Luminance, Motion, Movies, Narrative, Scenes, Shots

## Abstract

Popular movies grab and hold our attention. One reason for this is that storytelling is culturally important to us, but another is that general narrative formulae have been honed over millennia and that a derived but specific filmic form has developed and has been perfected over the last century. The result is a highly effective format that allows rapid processing of complex narratives. Using a corpus analysis I explore a physical narratology of popular movies—narrational structure and how it impacts us—to promote a theory of popular movie form. I show that movies can be divided into 4 acts—setup, complication, development, and climax—with two optional subunits of prolog and epilog, and a few turning points and plot points. In 12 studies I show that normative aspects in patterns of shot durations, shot transitions, shot scale, shot motion, shot luminance, character introduction, and distributions of conversations, music, action shots, and scene transitions reduce to 5 correlated stylistic dimensions of movies and can litigate among theories of movie structure. In general, movie narratives have roughly the same structure as narratives in any other domain—plays, novels, manga, folktales, even oral histories—but with particular runtime constraints, cadences, and constructions that are unique to the medium.

In any medium, a narrative can be thought of as a chain of events occurring in time and space and linked by causes and effects. . . . The basic principle of the Hollywood cinema is that a narrative should consist of a chain . . . that is easy for the spectator to follow. . . . The glory of the Hollywood system lies in its ability to allow its finest scriptwriters, directors, and other creators to weave an intricate web of character, event, time, and space that can seem transparently obvious.

—Kristin Thompson (1999, pp. 10—11)

No one need apologize for being interested in movies. Nonetheless, I recognize that their study is not an empirical psychologist’s usual professional fare. Movies are seemingly too complex—as complex as life, and often even more engaging. Here I explore the structure of popular cinema, analyzing 150 movies released across 75 years. The questions I wish to answer start with the structural and end with the psychological: What is the average film like? How might its presentational structure affect us? Might that structure provide keys to our engagement and perhaps even to framing new questions about our narrative comprehension?

Popular movies are stories, narratives. Narratology is the study of stories and story structure and the ways these effect our perception, cognition, and emotion. The everyday stories that we tell each other are the reconstruction of our experience in narrative form, and these become the units of remembered life. Moreover, finely tuned stories are the content of most works in many of our arts—theater, literature, and film; often poetry, art, and dance; and occasionally music.

Understanding narrative structure has occupied many disciplines across the humanities and social sciences. In this vein, psychologists have studied story grammars (Mandler & Johnson, 1977; Rumelhart, 1975) and related concepts like discourse (Kintsch & van Dijk, 1978), scripts (Schank & Abelson, 1977), and schemata (Brewer, 1985). The latter two are important here because as we grow and accrue more knowledge about how the social world around us works, we develop expectations about how events should unfold. These make comprehension vastly easier in a complex world. In movies the prototypic schema form is genre. As Bordwell (1985, p. 36) noted: “In a Western, we expect to see gunfights, barroom brawls, and thundering hooves even if they are neither realistically introduced nor causally necessary.” In this article, however, I wish to go twice beyond genre schemata. First, I will investigate the narrative structure of popular movies in general. Second, through investigation of the extrinsic physical norms of movies I will make inferences about how that narrational form should affect the viewer.

Notice my focus is on the story as it is physically told, not on the story as it is comprehended. I will explore how this story form has been “designed,” albeit tacitly across generations of filmmakers, to engage spectators. To do so, I need to import and discuss ideas from the humanities. My goal is a standard one for the initial phases of any investigation in the cognitive sciences: I hope to develop a system for describing the structure of movies as stimuli and incorporate the insights gained into theory development. This type of approach has been used many times—in the domain of discourse as launched by Kintsch and van Dijk (1978) and modified by Zwaan, Magliano, and Graesser (1995); in the ecological approach to perception as launched by James Gibson and pursued throughout his later career (see Cutting, 1993); and in many more.

## The *Fabula,* the *Syuzhet,* and Film Style

A useful distinction in this context is an old one to film. It comes from the Russian formalists (e.g., Shklovsky, 1925/1990)—the *syuzhet* and the *fabula* (see also Bal, 1985; Bordwell, 1985). These terms have numerous parallels. In the literature on text, Chatman (1980) used the terms of *discourse* and *story,* but for me the term *discourse* is too tied to the notion of conversation (which I investigate in Study 8) to be unambiguous here. Bortolussi and Dixon (2003, p. 98) reviewed many other such pairs, such as the telling and the told, *logos* and *mythos*, chronological events and causal events, expression and content, and the how and the what; and Cohn (2015) discussed narrative structure versus semantics. In addition, the film literature often discusses the plot and the story (see, e.g., Barsam & Monahan, 2013), but the former also has the connotation of synopsis, which is not my focus here. Finally, the film literature uses the term *narration* when discussing the syuzhet and *narrative* when discussing the fabula (e.g. Bordwell, 2008), but these can induce confusion because the former implies a narrator and a spoken voice-over, which is not my intent. Thus, although the narration/narrative distinction is important, I will also use the Russian terms because they are less freighted in English and, to be sure, their unfamiliarity allows me to bend them a bit to suit my needs.

The fabula is the story in all its semantic, chronological, and causal detail. Against most of film and literary theory, I believe we should acknowledge that there are actually two fabulas. The first is in the minds of the story makers and, after the telling, a second recognizable but condensed fabula is in the minds of story consumers. Among the story makers of film—the scriptwriters, directors, actors, cinematographers, editors, and more—the first fabula is a socially, but not wholly, shared complex web of ideas; it is not completely in the head of any single individual.^1^ Within the story consumer, the second fabula could be represented as a mental model (Johnson-Laird, 1983), or better, as a network of situation models (Zwaan & Radvansky, 1998). Moreover, it might be assembled through a structure-building framework (Gernsbacher, 1995) or through any number of other approaches (for a review, see McNamara & Magliano, 2009).

The first fabula is dimensionally complex—indeed, so much so that some would call it formless (Pier, 2003, p. 86n). It must be, and will have been, extruded through a temporal and sequential bottleneck of physical media to create the syuzhet. Yet the syuzhet is not necessarily ordinal in its temporal presentation of the story. Flashbacks, flashes forward, and parallel action (where two or more narrative threads are interleaved) are common in film and literature. And perhaps most importantly the *art* of storytelling, according to Shklovsky (Schmid, 2010, p. 178) is the in conversion of the fabula into the syuzhet*.* The fabula may be potentially interesting, ironic, or fantastic, but it is also artless.

For her own part, the spectator constructs a second fabula (roughly, con*fabula*ting it) by processing the syuzhet*.* As Bordwell (1985, p. 52) suggested: “The syuzhet . . . is the dramaturgy of the fiction film, the organized set of cues prompting us to infer and assemble story information.” Again, what is constructed in the mind of the spectator is not at issue here; it will vary with every story and a bit with every individual. Instead, my interest is in the organization of the surface form of movies, its “artfulness,” and in how that might aid the spectator’s understanding and affect her aesthetic experience.

The syuzhet (the narration) has cues and prompts that are presented in a particular film style, the filmmakers’ choices of cinematic devices. Film style concerns all aspects of the craft of filmmaking—editing, staging, lighting, sound, framing, focus, color, and more. Those aspects of particular interest here are the first five: editing (shot durations in Study 1, shot transitions in Study 2, the distribution of action shots in Study 9, and of scene transitions in Study 10); staging (motion in Study 3 and character introduction in Study 7); lighting (brightness in Study 4); sound (music in Study 5 and conversations in Study 8); and framing (shot scale in Study 6).

## Narrative theory in theater and movies

Popular movies are often bemoaned. With derogation and an occasional sneer, they are said to be formulaic. Yet as Thompson (1999) suggested in the opening epigram, movies shouldn’t be looked down upon. As a story form they can be fine art, emotionally absorbing, thought provoking, educational, and entertaining, all at once (see also Brewer & Lichtenstein, 1982). They can even be thought of as a kind of mind candy where we get to exercise our theory-of-mind faculties to predict and then find out what characters think (Levin, Hymel, & Baker, 2013; Zunshine, 2012)—a rare treat not typically so briskly and fully enabled in the real world. Moreover, movies seem to aid the development of this ability in children (Mar, Tackett, & Moore, 2010).

Nonetheless, in an important sense, popular movies *are* formulaic. The root of the term *formula* is *forma*, meaning a shape or mold. Narrative formulae are theories that describe the narrative states (in the fabula) that drive the sequential arrangement and presentational style of the events in the syuzhet. The overriding question becomes: Is there consistency in the sequential structure of the syuzhet in popular English-language movies? I will demonstrate empirically that this has been the case for the last 70 years and more, and, although I cannot present the evidence here, I would claim much of it applies to global cinema. This consistency can be said to reduce the cognitive load on the viewer; a formulaic movie can proceed rapidly because it is has been crafted to be easy to understand. The variations in these physical parameters can also drive the emotion and attention of the viewer. But what are these formulae? And, historically, where do they come from?

### Narrative structure: acts and turning points

[Drama] is complete, and whole. . . . A whole is that which has a beginning, middle and end. The beginning supposes nothing wanting before itself; and requires something after it; the middle supposes something that went before, and requires something to follow after; the end requires nothing after itself, but supposes something that goes before.

—Aristotle (Upton, 1746, pp. 67-68)

The craft of telling stories, arranging the syuzhet so that it is maximally effective, is probably as old as our species. Indeed, its emergence could be used as a defining moment for our speciation from our less human forebears (Boyd, 2009). Storytelling proceeded through millennia of oral tradition, then much later spread through plays and then through literature, so that by the time full-length movies came along a century ago, there was an extremely well-articulated notion of how to tell a story.

**Fig. 1:**
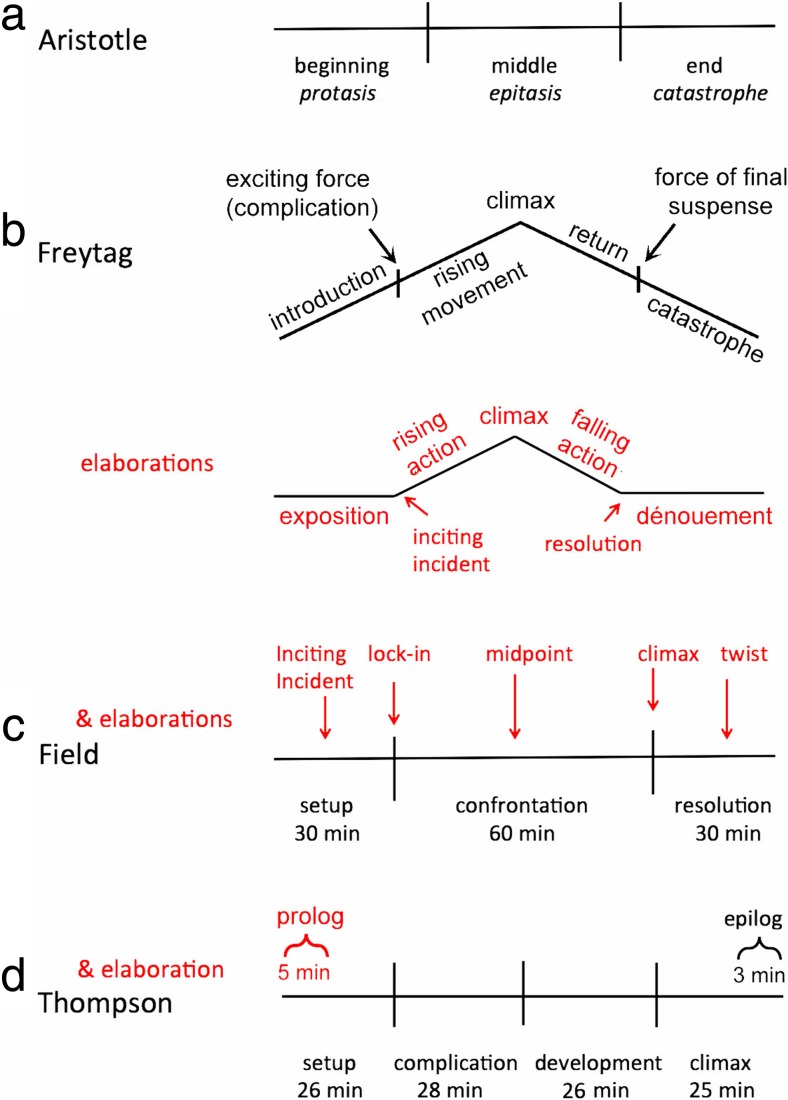
Four representations of narrative theories of literature and movies. Aristotle’s is based on his *Poetics* (Upton, 1746); Freytag’s comes from *Technique of the Drama* (MacEwan, 1900, p. 115); Field’s from *Screenplay: The Foundations of Screen Writing* (Field, 2005); and Thompson’s is based on *Storytelling in the New Hollywood* (Thompson, 1999). Elaborations in red come from others, cited in the text

The initial formulation of narrative structure is at least 2,500 years old and was promoted by Aristotle. In his oft-quoted and much-modified analysis, given above, he wrote that stories are wholes in three parts. More concretely, in Greek theater, the beginning of a play, the *protasis,* introduces the characters and setting; the middle of the play, the *epitasis,* contains the main action of the story building to a climax; and the end, the *catastrophe,* presents the climax and final resolution (Pavis, 1998), as schematized in Fig. 1a.^2^

### Theater in five acts

Later, in the first century BCE, however, a differing and more complex theoretical view arose. In his *Ars Poetica,* the Roman poet Horace suggested: “No play should be longer or shorter than five acts” (Kline, 2005, line 189). Indeed, there has been a long history of pentalogies (five-part structures) in literature and plays. For example, the Torah (*Penta*teuch) consists of the first five books of the Bible, and all of Shakespeare’s plays have five acts. In a flourish, the 19th-century English poet George Meredith (1897, p. 81) wrote that “five is dignity with a trailing robe; whereas one, two, or three Acts would be short skirts, and degrading.” Moreover, although American critic Brander Matthews (Matthews 1908) thought five acts a folly, he acknowledged that this pattern pervaded English, German, and French plays, but not the Spanish.

The five-part scheme was codified in the 19th century by the German novelist and playwright Gustav Freytag (MacEwan, 1900) with what is commonly known as Freytag’s pyramid, suggested in Fig. 1b. According to Freytag, these units began as separate, descriptive parts of drama—Roman theater was the first to use acts as a theatrical division, perhaps dictated by the needs for actors resting, changing costumes, or for scene changes—and then these became crystalized into separate functional, narrative units (MacEwan, p. 195). Freytag arranged them in an inverted-V shape (p. 115). Act 1 is the *introduction*, where characters and the setting are revealed and elaborated, followed by a narrative instant of the *exciting force* (or *complication*). Such an instant would later be called a turning point. Next is Act 2, the *rising movement*, throughout which the story builds in its impact. Act 3 is the *climax,* the pinnacle of narrative conflict, and Act 4 is the *return*, represented as an oblique line moving downward from the climax, where signs of the story’s end become apparent but where another turning point, the *force of final suspense* is necessary to keep the interest of the audience. Finally, Act 5 is again the *catastrophe.* The five-part, ordinally symmetric structure also seems to have some cross-cultural resonance. A 14th–15th century Japanese treatise by Zeami Motokivo describes the ideal form of the Noh play as having five acts (Yamazaki, 1984), with the third act having the greatest climax and the first and fifth being more peaceful and serene.

If we take the visual representation of Freytag’s pyramid seriously, however, it doesn’t really have five equal parts. The climax is represented as a high point in the middle, a turning point. The other four elements are lines that imply duration, even equal duration. Perhaps realizing this, several theorists have renamed and reshaped Freytag’s scheme (now with two flat wings and two oblique lines) into seven parts, with four larger elements and three turning points at their junctures (see Orr, 2003; Sternberg, 1978), also seen in Fig. 1b. In this modified version, the story begins with the *exposition*. Then, there is the first turning point, the *inciting incident*, which triggers the *rising action*. This continues until a second turning point, the *climax*, which is followed by the *falling action*, the final turning point of *resolution*, and the *dénouement,* where unresolved issues are cleared up.

Of course, given that the fabula and the syuzhet are concepts from the early 20th century, neither Aristotle nor Freytag could admit of such a distinction. But given that Freytag’s analysis of acts is about narrative states, it seems most appropriate to think of them as divisions of a generalized fabula.

### Movies in three acts

For lack of a better term, the largest narrative unit in movies has also been called the *act*, and I will follow that precedent here. Clearly borrowed from the theater, the term *act* has some unfortunate implications for movies. One is that there will be a pause in the action—curtain down, perhaps a bathroom break, and in earlier times, a smoke and a drink in the lobby. Such breaks rarely happen in the popular movies of the West, except most glaringly in the long epics of the 1960s and 1970s (e.g., *The Sound of Music*, 1965; *Patton*, 1970), when there was an intermission about three-fifths through the movie. Moreover, the last thing any contemporary filmmaker would want is for the filmgoer to leave the theater and go buy popcorn while missing some of the action. Large-scale continuity aspires to have viewers stay riveted to their seats, wanting to know what happens next in a continuous flow. Nonetheless, I claim that movies do have natural joints at which the narrative (fabula) can be carved, and the goals of this paper are (1) to understand and empirically demonstrate how those moments, and the acts that they bind, are physically instantiated (in the syuzhet) and (2) to begin to suggest what psychological consequences these might have.

However much the five- and more-part narrative schemes may have influenced literary studies, they haven’t influenced screenplays or film studies. Perhaps this is because movies differ from plays and literature, particularly in their temporal constraints. Bladder control is an issue for uninterrupted movies that might run much longer than 150 minutes, and the practical economics of the pre-Cineplex era dictated that such movies were awkward to present in two showings after dinner. Moreover, although the ordered relations of the literary schemes may make some sense in the filmic context, as applied to the runtime of movies they are deeply problematic. For example, the dénouement (or epilog) of a movie is almost always only a few minutes long, whereas anything that might be called the rising action can take up more than an hour.

Aristotle’s view, rather than Horace’s or Freytag’s, is the intellectual forebear of most analyses of movie narratives. Although some earlier accounts of screenwriting suggest flexibility in realizing Aristotelian form (see Nash & Oakey, 1978), the most commonly promoted three-act approach to screenwriting is laid out in quite rigid runtimes, as sketched in Fig. 1c (Field, 2005; see also Aronson 2001; Egri, 1942; Fridja, 1986; Lavandier, 2005; but see Brütsch, 2015, for a skeptical view). In Field’s analysis, the first quarter of a movie is called the *setup* (Act 1, about 30 min in duration), the next half is the *confrontation* (Act 2, about 60 min), and the last quarter is the *resolution* (Act 3, about 30 min).

There are myriad variations on this three-act theme, and it would be tedious to explicate them here. It will be useful, however, to discuss one, but to do so I must first define two terms suggested in their use by Thompson (1999, p. 23). First, *turning points* are narrative moments from which the plot goes in a different direction. By definition these occur at the junctions of acts. Second, *plot points* are important events in the narrative that can occur anywhere, but to maintain them as different from turning points I will define them as occurring within an act.

One fairly intricate three-act narrative scheme that goes beyond most other elaborations is found on the Scriptlab website (http://thescriptlab.com), codified by a consortium of scriptwriters. It presents an eight-part description of cinematic narration: it has two turning points at junctures of the three acts, and three plot points, one within each act, as suggested in Fig. 1c. In narrative order these are: (1) the *inciting incident*, here about halfway through Act 1 and not at the juncture of Acts 1 and 2 as in the elaborations of Freytag’s scheme; (2) the *lock-in* at the transition between Act 1 and Act 2, where the protagonist becomes committed to action; (3) the *midpoint* of Act 2, or the first culmination, where the protagonist experiences her first success or failure; (4) the main culmination, or *climax,* at the end of Act 2, where the final conflict for the goal begins; and (5) the *twist* in the middle of the resolution (Act 3), where narrative events take a turn and change the tenor and interpretation of the plot.

It is by no means clear how many movies follow the Scriptlab formulation—twists do not seem ubiquitous, although they do have some resonance with Freytag’s *force of final suspense*. Nonetheless, some movies almost certainly have such a form. But I believe that the more complex the narrative scheme, the less general it is likely to be. Furthermore, I suggest that one should seek a scheme that is relatively simple, that has a narrative form (in the fabula) with physical implications (in the syuzhet), and articulated enough so as to be testable.

### Movies in four acts, and some data

Thompson (1999) and Bordwell (2006) proffered a four-act elaboration of Field’s structure. The main change is that the central act (Act 2) is divided in half. After all, this act is often said to have a midpoint that separates the two parts. Perhaps the notion of rising action until the climax has obscured changes in the narrative that come between it and the setup. Or, as Thompson suggested, the sections of setup and climax are obvious, but what goes on in between is much less so. Thompson would acknowledge turning points between acts, but she chooses, perhaps wisely, not to name them (but see Bordwell & Thompson, 2011, pp. 126–134, for a taxonomy of types). In addition, the last act and perhaps also the first have their own subdivisions without particular time constraints. The result is that each of the four acts lasts about one quarter of the runtime of a movie, typically a bit less than a half hour, as suggested in Fig. 1d.

In this four-act scheme the *setup* is first, as it is in Field’s (2005) analysis. Again, the viewer is introduced to the characters, their goals, and the environment in which they find themselves. The setup is followed by the complicating action, which I will simply call the *complication*. Here the protagonist’s goals are derailed, she realizes that her tactics must be reformed, or she finds wholly new circumstances forced upon her. Third is the *development*, beginning at the midpoint of Act 2 in the Field scheme, where the story typically broadens, often as subsidiary characters play a greater role and while the protagonist struggles but remains fully committed to her goal. And fourth there is the *climax*, where the protagonist finally launches, or is launched, into action. Most movies also have a short *epilog* at the end of the climax section—signaled by an unnamed plot point that I will simply call *the end.* After this the new normal is established, and loose ends of the plot are tied up. Such restorations of a social order have been a part of discussions of narrative form, at least since Tillyard (1942).

In support of this scheme, and to show that it is widely and historically applicable, Thompson (1999) analyzed the narrative structure of 10 movies in great detail, and 100 films across 100 years more, briefly. Thus, three aspects of Thompson’s approach make it more attractive and empirically more substantial than others. First, she sought statistical regularity through the evaluation of a large sample of movies rather than relying on cherry-picked case studies, the technique that dominates this literature.

Second, with Nash and Oakey (1978), she noted that no narrative organization should be considered procrustean. Different movies will have somewhat longer and shorter acts as the presentation of that narrative requires, or at least as it has been developed over revisions of the screenplay. Indeed, Thompson’s analyses provide the following data across her sample of 73 four-act movies: the setup averages 26.2 min (24.9% of average runtime of movies in her sample, excluding text-only opening credits; standard deviation (σ) = 2.8%), the complication averages 27.8 min (26.5%, σ = 3.7%), the development 26.3 min (25.1%, σ = 3.5%), and the climax 24.6 min (23.5%, σ = 3.8%). Finally, epilogs, which appear in 83% of Thompson’s sample, have mean duration of only 2.4 min (1.8%, σ = 1.6%) contained within the climax. Movies without epilogs include *Three Days of the Condor* (1975), *Sleepless in Seattle* (1993), and *You’ve Got Mail* (1998). All mean values are shown in Fig. 1d.

Third, Thompson noted that the four-act scheme must be modified for movies of nonstandard lengths. Movies longer than 150 min may have five or more acts, often with multiple development sections. Movies between 60 and 90 min might have three or four acts, although most of those that Thompson analyzed have four. Finally, movies less than 60 min may have only three acts, often skipping a development section.

Thompson conjectured that something might be psychologically pertinent about balanced narrative sections of 20 to 30 min in duration (Thompson, 1999, pp. 43–44). Bordwell (2008, p. 104), on the other hand, wondered if equal-length acts might be a carryover from equal-length reels (~15 min in length) in earlier cinema, where pauses between reels forced filmmakers to organize plot structure so that scenes were sufficiently complete but outcomes insufficiently known to keep viewers in their seats wanting more while projectionists rewound and remounted the reels.

Thompson’s analyses are intriguing, but, despite her close readings of movie narratives, psychologists would typically call her data subjective. That is, they are based solely on her parsings as a film scholar. Thus, with the existence of other schemes and a panoply of variations across the literature, it is difficult to litigate among theories without independent and more empirical data.

### The major assumption: the syuzhet should reflect narrative states

Why should the number of acts matter? Aside from seeking descriptive adequacy, I assume that if any one of these narrative theories is generally correct, then there should be characteristic physical changes in the style of movie presentation as their stories unfold. More broadly, I assume that, at least in the domain of popular movies, *theories of the fabula must predict physical consequences in the syuzhet;* and reciprocally*, aspects of variation in the syuzhet should reflect the structure of the fabula.*

Why should I assume this? Basically, my view is an extension of Bordwell (1986, p. 26): “Classical narration treats film technique as a vehicle for the syuzhet’s transmission of fabula information.” The extension here is to apply this idea to the whole length of movies and serially in one stylistic dimension at a time. Given a tight relation between fabula information and syuzhet technique, there must surely be some manifestation of the fabula in the surface form throughout a standard, popular movie. How that form might be revealed was unknown to me as I began these studies, but I had a firm commitment to exploring how it might be manifest.

Why is this important for psychology? First, results here show that aspects of the surface form and aspects of meaning are not independent, at least not in the domain of popular movies. The relation between form and meaning has been of continual interest in philosophy, psychology, and linguistics at least since Saussure (1916/1949) and Ogden and Richards (1923). Second, for completely understandable reasons, previous psychological work on textual narratives (e.g., Mandler & Johnson, 1977; van Dijk & Kintsch, 1983; Zwaan, Langston, & Graesser, 1995) has focused on rather short stories, rarely more than about 1,000 words. Here, I have the opportunity to look at the interrelation between narration and the narrative in many, much larger sources to assess differences between smaller scale and larger scale structures.^3^

## General methods

### A sample and subsample of movies

I explore the possible physical ramifications of movie narratives in a broad corpus of movies. My larger sample initially contained 160 English-language films released at 5-year intervals between 1935 and 2010, 10 per year.^4^ These movies were culled from five different genres—32 action films, 22 adventure films, 12 animations, 43 comedies, and 51 dramas. Again, across these I will be searching for a general formula of narrative form, not a genre-specific one.

All movies were chosen from among the highest grossing or the most widely rated movies of their given release year, as posted on Box Office Mojo (http://www.boxofficemojo.com) or the Internet Movie Database (http://www.imdb.com), under the condition that at least four of the five genres be sampled (not every such year had a successful animated movie). The 150 movies from 1935 to 2005 are listed in the supplemental material to Cutting, DeLong, and Nothelfer (2010); the additional 10 from 2010 are in the appendix to Cutting, Brunick, et al. (2011a, b). When analyses on the larger sample demanded too much human research effort, I dropped back to a subsample of 24 of these movies—one action film, one comedy, and one drama released every 10 years from 1940 to 2010. This subsample of movies can be found in the appendix to Cutting, Brunick, and Candan (2012). At 24 frames/s, the movies in both samples averaged about 165,000 frames.

Because my focus is on movies that are likely to have four acts in Thompson’s (1999) scheme, the larger sample reduces to 150 movies and the smaller sample to 23, culling out 10 movies longer than 2.5 hours and those with intermissions. Excluding final credits, the remaining movies have runtimes that vary from 61 min (*Westward Ho*, 1935) to 147 min (*The Color Purple*, 1985). If the opening credits are superimposed on early shots, a stylistic procedure that began a bit before 1960, then these shots are included in runtimes; otherwise, if opening credits are a series of title cards (shots of unmoving text), they were not. I will call these two groups overlaid- and trimmed-credits movies, respectively.

### New analyses of previous data, New data, and ordinal time

I began with aspects of data gathered previously about shot durations (Cutting, DeLong, & Nothelfer, 2010; Cutting, DeLong, Brunick, Iricinschi, & Candan 2011b); shot transitions (Cutting, Brunick, & DeLong, 2011b, 2012); motion (Cutting, Brunick, & DeLong, 2012; Cutting, DeLong, & Brunick 2011a); luminance and music (Cutting, Brunick, & Candan, 2012); shot types (Cutting & Candan, 2015); and narrative shifts from one scene to the next (Cutting, 2014; Cutting & Iricinschi, 2015), although some of these were recalculated for this set of studies.

My method of exploring movies concerns one aspect of what in the humanities would be called *temporality* (e.g., Drucker, 2011). In particular, I am interested in how information unfolds over a movie’s narration (its ordinality, or sequence in time) rather than with a strict meter (metric time). Thus, unlike in most of my students’ and my earlier work, I have affine transformed the duration of each movie by dividing them into equal-duration, consecutive time bins.

For most analyses I chose 100 bins. Thus, if a movie was 100 min long, each bin was 60 s in duration; if it was 65 min long, each bin was 39 s, and so forth. Within those bins I derived the average shot duration, tallying the number of cuts and other transitions (Study 1); I recalculated the amount of motion, and brightness of the shots (Studies 3 and 4); and I used previous data on whether music occurred (both diegetic and nondiegetic music–that heard by the characters or only by the audience, respectively), on how different shot scales are used, and on when new characters and locations are introduced (Studies 5, 6, and 7). When data were more sparse, I did coarser analyses in 20 equal-duration consecutive time bins for noncuts—dissolves, fades, wipes, and other nonstandard transitions (Study 2); for shot types (Studies 8 and 9); and for narrative shifts across locations, characters, and time (Study 10). In most cases data were then normalized by movie and medians taken across movies. None of my previous articles, except Cutting, Brunick, and DeLong (2012), performed this kind of analysis, and all of what I present here is new.

### Analytic style

Again, the data were gathered for each movie and aggregated such that each contributed equally. For most studies I present a plot of the movie medians for 100 or of 20 consecutive equal-duration bins of data. This is not to assume that movies do not differ from one another or that they have not changed over time; they do and have (Cutting & Candan, 2015; Cutting, DeLong, & Brunick 2011a; Cutting, DeLong, Brunick, Iricinschi, & Candan, 2011). Nonetheless, those differences and changes are not the focus of this investigation, and they are generally removed by normalization (but see Study 9).

I analyze the data in the first 10 studies by fitting linear, exponential, but mostly polynomial regression lines and assess the best fit. I fit polynomials under the assumption the changes in the aggregate data occur relatively smoothly, in part because I am averaging across movies with differently proportioned acts (not always exactly one quarter of the film length). I assume that the extrema or inflection points of the functions should occur at turning points and plot points according to film theory.

Perhaps most importantly, this work is unabashedly exploratory. That is, all of these studies were undertaken with the expectation that there should be some physical trends (in the syuzhet) that correlate with the structure of the narrative (the fabula), but with little preconceived notion of what those trends might be. To justify the idea of presenting a *theory* of the narrative form in popular movies, then, I needed to replicate central results. In the context of these corpus analyses, the best method is cross-validation. That is, after finding the global pattern in the data of the densest datasets, I then divided the movie samples into many random halves, ran the analysis on a given half as a training sequence to derive the parameters of fit, and then used those values in a parameter-free assessment of the test halves. Good fits in both training and test halves are good evidence for reliable and believable patterns.

### Terms

*Shots* are continuous runs of image frames that create motion. A *cut*—the abrupt frame-to-frame switch from one shot to the next—forms the most common transition between shots. Indeed, almost 99% of all shot transitions in contemporary movies are cuts (Cutting, Brunick, & DeLong, 2011b). Noncuts include *dissolves* (a transition typically over 20 or more frames that gradually blends from one shot to the next), *fades* (dissolves that go to black or to some other color before fading in to a new shot), *wipes* (where one shot is replaced by the next across a moving boundary in the frame), and several other exotic transitions. These noncuts typically occur at narrative breaks in a movie, often moving from one scene to the next. Historically, these transitions signaled a scene change and an ellipsis in time. But dissolves also often signal a change in tone, performing a calming effect on narrational pace.

*Shot scale* refers to the relative size of a character within the frame. This is a continuous variable that is typically divided into seven categories: extreme long shot (1, a wide angle shot of an environment, often with a character in it but which extends well beyond her feet and head), long shot (2, where the feet and head of the character are barely within the frame), medium long shot (3, cutting the character at the knees, which the French have called *le plan americain*), medium shot (4, cutting the character at the waist), medium close-up (5, cutting the character at midchest), close-up (6, full face but cutting the character at the shoulders), and extreme close-up (7, showing just the face or only part of the face of a character). This scheme easily extends to other body parts (hands) or to familiar objects (refrigerators, keys, or smart phones) scaled to the size of a person.

*Narrative shifts* occur when the movie proceeds at a transition from one *scene* to the next.^5^ These can occur for at least one of several reasons: a shift in location, a shift in characters, or a shift in time—or any combination of these. Potentially, and on most occasions in a movie, viewers will recognize that a change in one or more of these dimensions signals a change in one or more of three narrative units (Cutting, Brunick, & Candan, 2012). These units are *sequence, scene*, and what we call *subscene*. In film theory, Bellour (1976) called them *suprasegments, segments,* and *subsegments,* respectively.

A *scene* can be defined as taking place generally within one location, with a single set of characters, across a contiguous span of time. A *subscene* is a convenient unit that often occurs within sequences of parallel action. A *sequence* typically occurs when there is continuity of time that spans several scenes or subscenes, but with no constraint on whether characters or locations shift. For example, in an action sequence, the protagonist and antagonist may be moving toward a common goal, and the narrative alternates between subscenes across a continuous time frame. A subscene, like a scene, takes place in a single location and time frame, with a single set of characters, but it is typically shorter, often with only a few shots, and it may have no obvious beginning or ending—that is, its content is fully comprehensible only in the context of a previous scene or subscene and will be resolved only in a subsequent one. However, in what follows, I will collapse over differences between scenes and subscenes.

Finally, most scenes begin with an e*stablishing shot.* This shot sets up a scene, typically with longer duration and scale that places characters within an environment. What I will call a *reestablishing shot* is one where the syuzhet returns to a location that has been seen before. In film literature, however, such a term is usually used for a *two shot* (showing two characters) in a longer conversational scene after a series of *shot/reverse shots*—showing one character and then the other in dialog. Its purpose is to refresh the memory of the viewer about where the characters are and often to denote a change of tone in the discussion.

### Twelve studies in four parts

These studies are broken into four parts, and the order in which I present them is driven by pragmatic (indeed storytelling) concerns. Part 1 contains the first four studies that present the strongest results, are based on the most data, and provide the clearest evidence for parsing the syuzhets of movies into four acts based on physical parameters (changes in shot durations, Study 1; changes in noncut transition frequency, Study 2; changes in motion, Study 3; and changes in luminance, Study 4).

Part 2 is a residual. It contains the two studies that show physical aspects of syuzhets that are quite independent of the acts (music, Study 5; shot scale, Study 6). These show that filmmakers tacitly create syuzhets, shaping them at a minimum of three levels of narration—the whole movie, the acts within the movie, and the scenes typically within the acts. Although the focus of this article is on this middle level, considerable flexibility of narrational form is gained through the independence of these levels.

Part 3 contains four studies that investigate more directly some functional aspects of the narrative and less the auditory or visual narration. These include when protagonists are introduced (the setup, Study 7), when characters converse (the complication and the development, Study 8), when action shots occur that might distinguish genres (mostly in the climax but with some surprises elsewhere, Study 9), and when and how scenes change (Study 10).

And finally, Part 4 melds the first 10 studies with a componential analysis (Studies 11 and 12), unifying the results, placing them within four correlated narrational dimensions and a fifth that is independent. Nine of these results fit into two orthogonal components of the syuzhet, and the 10th in its own component.

## Part 1: Variations in film style across and within acts

### Study 1: How shot durations pace narration

A movie, I think, is really only four or five moments between two people; the rest of it exists to give those moments their impact and resonance. The script exists for that. Everything does.

—Robert Towne (cited in Howard & Mabley, 1993, p. 3)

Every cut has the potential of forcing a viewer to reallocate attentional resources (T. J. Smith, 2012). Partly as a result of this, mean local shot duration can be taken as one measure of the relative local intensity of a movie (Bordwell, 2006; Cutting, DeLong, & Brunick, 2011a; Cutting & Candan, 2015). Fewer cuts and longer duration shots typically reflect calmer and more expositional sections of narration (the syuzhet); more cuts and shorter duration shots typically reflect more conflict and action (see also Pearlman, 2009). Given this relation it seems reasonable to assume that the generalized pulse of movie narration (the syuzhet) should be found in the pattern of shot durations that extend through it. This pattern might also frame the narrative moments, perhaps turning points, suggested by Academy Award winning screenwriter Robert Towne (*Chinatown*, 1974) in the preceding epigram.

#### Methods

Regardless of the number of transitions in a given movie—ranging from just over 200 to just under 3,000 in the large sample–I normalized the data so that their sum in each movie added to 100. Thus, for a movie with 500 transitions, each transition within a given bin counted 0.2; for a movie with 2,000 transitions, each counted 0.05, and so forth. Because there are 100 bins, there is necessarily a mean of one normalized transition within each bin.^6^ In this manner and reciprocally, the fewer the transitions, the longer the local durations of shots; the more transitions, the shorter the shots. More importantly, every movie contributes equally to the pattern of results.

#### Results and discussion

**Fig. 2:**
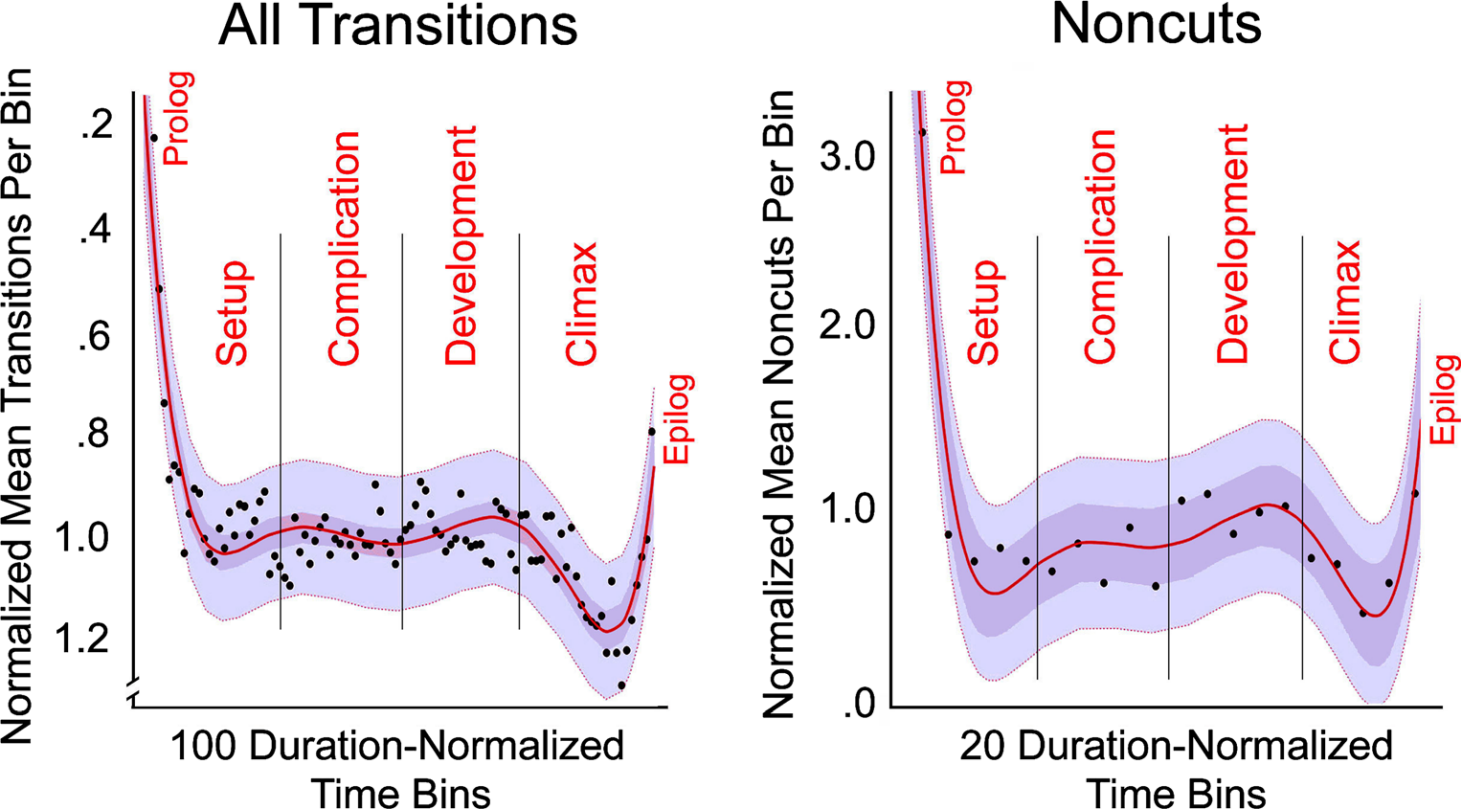
Median densities of transitions across the normalized duration of the large sample of movies. The left panel shows the results of 150 movies in Study 1, where the densities of all shot transitions (about 88,000 and predominantly cuts) are normalized across 100 consecutive, equal-duration bins for each movie. The right panel shows the results of 148 of those movies in Study 2 for dissolves, fades, and wipes (about 5,500), only across 20 such bins. Notice that the patterns are essentially the same, but the ordinates are reversed. Two confidence intervals are shown: a 95% interval on the regression lines in darker purple, and a 95% interval on the data in lighter purple

The left panel of Fig. 2 shows a plot of the median density of shot transitions across the 150 films in the large sample. Notice that the ordinate of the graph is inverted so that the inference to longer shot durations has them going up the scale. Running through the bin medians is a smooth sixth-order polynomial fit.

The complexity of this function might seem outrageous for a regression line. What theoretically legitimizes it is that it can fit seven points of interest—five that could be local maxima or minima and two endpoints. Indeed, following narrative theory, I wished to fit seven points that might be spread over the space defined by shot duration and by the runtime—the two endpoints, the very beginning and the very end of the movies or Bins 1 and 100; and as many as five points suggested by Robert Towne. These include the three in the vicinity of Bins 25, 50, and 75, marking potential boundaries among the four acts; and two floating plot points of articulation, one for a possible inciting incident (roughly between Bins 10 and 20) and the other for *the end* at the beginning of the epilog (roughly between Bins 95 and 100). Corroborating the use of this multiparameter characterization, the plotted function is the best polynomial fit to the data, adjusted *R* ^2^ = .74, *F*(6, 93) = 48.4, *p* < .0001, reliably superior to the fits of all lower order polynomial functions (where adjusted *R* ^2^s = .57, .57, .39, .25, and .24 for the fifth-order polynomial on down).

Despite the statistics and the visual salience of this polynomial fit, one should be a bit skeptical. Thus, to ramify and reinforce it, I performed multiple two-fold cross-validation tests to assess the robustness of this pattern. To this end, I randomly divided the 150 films into two sets, a training set and a test set of 75 films each, and did so 10,000 times. In each case I took the median values across the 75 movies for the 100 bins and fit the training set with a sixth-order polynomial. The training fits yielded results reasonably close to that for the whole set (median adjusted *R* ^2^ = .60) and the test set fits were not much inferior (median *R* ^2^ = .56),^7^ suggesting very modest overfitting. Median root-mean-square deviations (RMSDs) for both sets were 0.149 and 0.163, respectively. There was no effect of release year, and the only effect of genre was that action films showed a stronger commitment (*R* ^2^ = .51) to the sixth-order polynomial than did dramas, *R* ^2^ = .33, *t*(81) = 6.07, *p* < .0001.

This cross-validation result gives me some license to look at the local aspects of the pattern shown in the data. Mean shot durations are very long during the first part of the setup, when filmmakers introduce the story, the characters, and the environment. These long-duration shots bring the viewer into the story in a deliberate and inviting way. Reasonably quickly, however, the shot durations regress to their mean (transition density near 1.0 per bin), likely in a normal progression from an establishing exposition to the narrative proper and its dialog. Dialog shots typically have about the same average duration as those of the entire movie (Cutting & Candan, 2015). This latter point largely results from the fact that more than half of all shots in the movies in our smaller sample show dialogs, as I will explore further in Study 8.

Median shot durations are longer in the setup than in the complication, mean transition densities = 0.92 versus 1.00, *t*(48) = 8.50, *p* < .0001, *d* = 2.32, even if one omits the data in the first two bins, considering them as outliers. This result implicates a slower pace in the setup that changes at the lock-in. Shot durations then lengthen again slightly from the complication to the development, transition densities = 1.00 versus 0.98 per bin, *t*(48) = 2.18, *p* = .034, *d* = .63. In the development the narrative typically gets more complex, often dodging back and forth across parallel subplots. The establishing and reestablishing shots, which open new scenes and subscenes, are typically longer in duration than the average shot (Cutting & Iricinschi, 2015). Shot durations fall precipitously during the first part of the climax. This is where physical action is typically greatest. And finally, they rise smartly during the epilog, when diegetic world order is generally restored.

One might suspect that action films contribute mightily to the dip in the climax, but they do not, and for two reasons. First, only 30 of these 150 movies are action movies, so it seems unlikely that they could statistically dominate; and second, the typical action film usually has a number of action sequences peppered throughout the narrative (as I will discuss in Study 9). These apparently leave few if any traces in these overall data. Thus and instead, the trend in the left panel of Fig. 2 should be considered characteristic of the syuzhets of all genres.

The data are a bit noisy to be sure, but I find it remarkable that there is any trend at all, and that the trend makes narrative sense. Again, these movies vary in length from an hour to almost two and a half hours. And remember, this analysis assumes nothing other than a unit length of movies, shots proportioned by the locations of transitions across that length, and four equal-duration acts marked off along its course.

In sum, these data offer reasonable support for a four-act theory of movie narrative with points of articulation within the first and last acts. That is, under the assumption that the physical pacing of movies, as measured by shot durations, is an index of the intensity of the narration (Bordwell, 2006), one can see that movies start slowly for expositional purposes, speed up to a normal narrational progression, slow down slightly as the narration gets more complex, rush through the climax, and then back off as normality returns. What other evidence might be allied with this structure?

### Study 2: How noncut transitions accentuate the pace of narration

If there is a comma in film amongst this catalogue of periods [shot transitions], it is the dissolve. . . . It is the one mark of punctuation in cinema that mixes images at the same time that it conjoins them.

—James Monaco (1977, p. 192)

Consider parallel evidence in the density of all transitions that are not cuts—the dissolves, fades, wipes, and other noncuts, with dissolves by far the most common. Cutting, Brunick, and DeLong (2011b) noted that the use of noncuts declined dramatically in popular movies after about 1965, but they have not entirely gone away. Noncuts occur in contemporary movies between all shots about 1% of the time, and between shots of different scenes about 10% of the time. Moreover, they represent about 5,500 of the 188,000 transitions in the larger movie sample. Thus, there are ample data here to exploit.

#### Methods

Of 150 movies in the larger sample, two (*MASH*, 1970 and *Die Hard 2*, 1990) had no dissolves, fades, or wipes, so 148 movies are represented in the analyses to follow. As with the all-transitions data underlying the left panel of Fig. 2, the noncuts in the right panel were normalized within each movie, summing again to 1.0 per bin, but here, because of the relative sparseness of the data, there were only 20 equal-duration bins rather than 100. In this manner, if a movie had 50 dissolves and fades, each would count 0.4 in a given bin; if a movie had only 10 noncuts, each would count 2.0.

#### Results and discussion

Notice in the right panel of Fig. 2 that the general pattern of noncuts across the 20 bins parallels that for shot durations seen in the left panel. It is again fit with a sixth-order polynomial, adjusted *R* ^*2*^ = .87, *F*(6, 13) = 22.05, *p* < .0001, it is again superior to all lower order fits (adjusted *R* ^2^s = .71, .71, .38, .16, and .06), and in 10,000 two-fold cross-validation tests the sixth order fits to the training and test data (median adjusted *R* ^2^ = .81, and median *R* ^2^ = .70, and RMSDs of .246 and .384, respectively) suggest that this polynomial is a reasonable expression of the underlying pattern of the syuzhet.

But consider: This pattern is the opposite of what one might expect according to the results of Study 1. Indeed, grouping the data from the 100 bins of the first panel into 20 equally spaced bins and comparing the two datasets, the correlation is negative and striking, *r* = -.89, *t*(18) = -7.86, *p* < .0001, *d* = 3.7. That is, higher values in the left panel indicate *fewer* transitions of all kinds, whereas higher values in the right panel indicate *more* noncut transitions. Thus, as transitions become fewer in the narrational stream, dissolves, fades, and wipes become more prevalent.

The dissolve, in particular, has many functions. It is used as a simple transition from one scene to the next, it is used to show a change in time, it is used to slow down the narration or to change tone, and it is used among shots in a montage (Cutting, Brunick, & DeLong, 2011b; Monaco, 1977). Notice, these noncuts occur commonly at the beginning of the setup, where there may be an early montage sequence. They then decline by its latter half (and with no significant change across the lock-in) and through the complication when the narration progresses and halts or slowdowns might be disruptive. They rise again during the development, where again the syuzhet often crosses between narrative threads, (0.80 noncuts per bin in the complication versus 1.08 in the development, *t*(8) = 2.78, *p* < .012, *d* = 1.97. Their number then declines to its lowest relative frequency during the climax, where fades and dissolves would impede the action.^8^ Finally, the frequency of noncuts climbs during the epilog, when diegetic social order is typically reestablished and narrative threads are resolved.

### Study 3: Narration and the rollercoaster of motion

The term *cinematography* is from the Greek roots meaning “writing with motion.” . . . It is the process of taking ideas, words, actions, emotional subtext, tone, and all other forms of nonverbal communication and rendering them in visual terms.

—Blain Brown (2012, p. 2)

All creatures with eyes respond to motion. Motion can signal the necessity for action; it can also prime us for action through a cascade of physiological responses. Increases in motion can affect skin conductance, pupillary responses, and heart rate; all three of these are correlated with emotional responses; and all are triggered by motion in movies (Ando et al, 2002; Carruthers & Taggart, 1973; Soleymani, Chanel, Kierkels, & Pun, 2008). Indeed, it is often said that the most important goal of editing is to control the emotions of the viewer (Murch, 2001; E. S. Tan, 1996). Thus, unsurprisingly given the etymology of the words, motion affects emotion.

In describing movie narration (the syuzhet), Keating (2011, p. 89) noted “the favoured metaphor is . . . [the] roller coaster, taking the spectator on a ride where his or her emotions are constantly swinging up and down.” Motion as it is distributed across the duration of a movie, then, is a prime venue to look for data that might corroborate a general theory of cinematic narrative form.

#### Methods

Motion can be measured in many ways (Borst & Euler, 2011). In general, however, the various kinds of motion are correlated with one another in naturalistic situations and in movies (Nitzany & Victor, 2014). Thus, I used the simplest method to compute motion. I started with the eight-bit luminance values (0 to 255) of all pixels in each frame, and then correlated those values in successive frames within each shot and then along the length of each movie. In this manner, a value of 1.0 means no motion, and decreasing values indicate more and more change across the images and, hence, more motion.^9^ This change is called *flicker motion* because it measures how much each pixel may “flick” on and off (or to higher and lower values of luminance) without tracking the correlated structural patterns of those pixel changes across space.

I had downsampled each frame of each movie to 256 × 256 pixels (about 65,500 pixels),^10^ converted the films to grayscale, and then ran a MATLAB script to correlate successive frames (except those across cuts) along the length of the movie, again averaging about 165,000 frames. As before, I divided each of the movies in the large sample into 100 equal-duration bins and averaged the within-shot pixel correlation values in each bin. Within each movie I then normalized bin values (mean = 0, standard deviation = 1) and took the median across movies.

#### Results and discussion

**Fig. 3:**
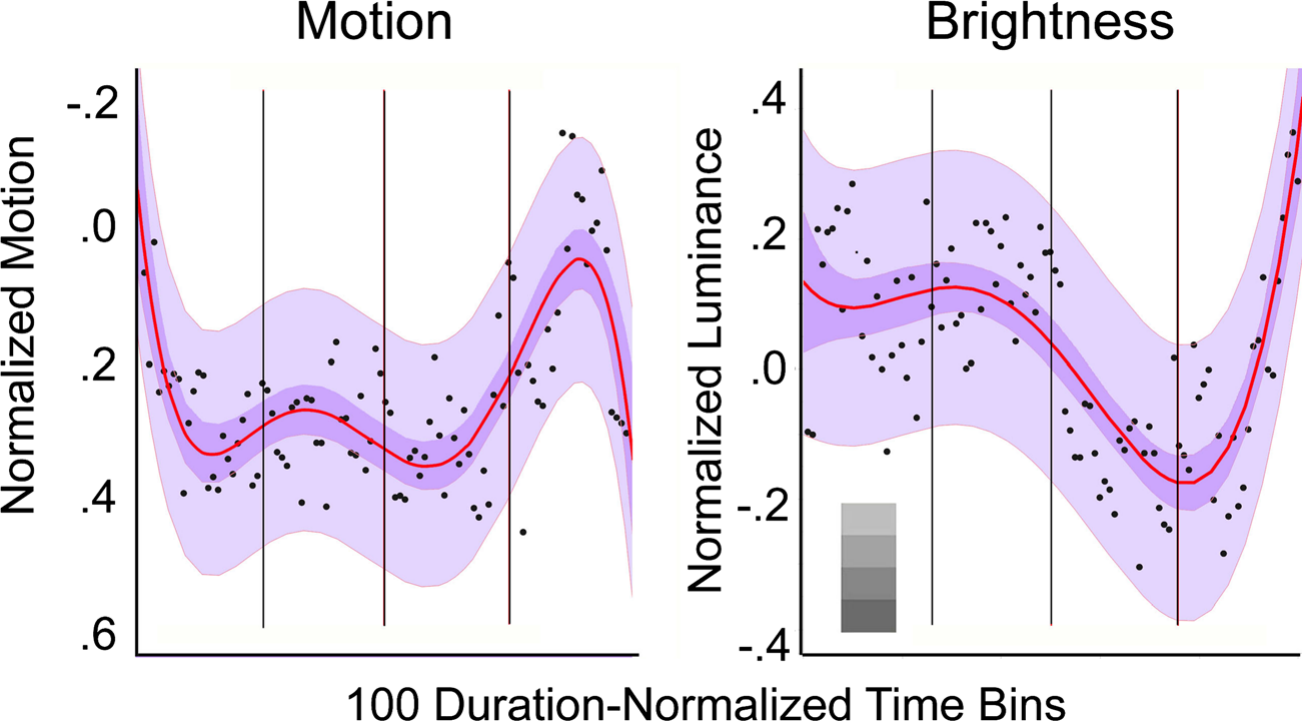
The distributions of normalized median motion (Study 3) based on across-frame correlation values and of normalized median luminance (Study 4), measures based on gamma-corrected luminance pixel values. Both are plotted across 100 duration-normalized bins for the larger sample of 150 movies. Both panels show polynomial fits to all of the data, and 95% confidence intervals are shown for the regression lines and for the data in darker and lighter purple, respectively. In the right panel, the difference between the mean values from the epilog (Bins 98–100) and nadir of the development (Bins 74–76) is about one step in the grayscale display shown at the lower right. Vertical lines separate the setup, complication, development, and climax, as in Fig. 2

The data are shown in the left panel of Fig. 3, and they are a veritable profile of Keating’s (2011) rollercoaster. This time a fifth-order polynomial is fit to the data, adjusted *R* ^2^ = .53, *F*(5, 93) = 23.1, *p* < .0001, it is statistically superior to all lower order fits (adjusted *R* ^2^s = .33, .32, .32, .12), and the 10,000 assessments of two-fold cross-validation yielded reasonably comparable results for training and test sets (median adjusted *R* ^2^ = .34, and median *R* ^2^ = .24; RMSDs = .127 and .142, respectively). Again, there was no main effect or interaction concerning release year, and only dramas and action films differed, *t*(81) = -5.62, *p* < .0001, with the latter conforming more closely to the pattern in Fig. 3.

The overall trend, particularly in the last part of these movies, is quite salient. There is a bit less going on in the motion domain during the first three-quarters of the average movie than later. Motion declines at the beginning of the setup, is generally stable through the complication and the development, and there is then a considerable swing of increasing motion through the climax and then a sharp return to general calm during the epilog. The quadratic trend in the epilog is quite salient, adjusted *R* ^2^ = .21, *F*(2, 23) = 4.43, *p* < .02. This would be allied with effects of shot duration and prevalence of dissolves, both shown in Fig. 2.

### Study 4: Narration’s brightest and darkest moments

Lighting shapes the reality in front of the lens, giving it depth or flatness, excitement or boredom, reality or artificiality. The art of cinematography is the art of lighting and making that light tell the story.

–Sam Kiwan and Leal Butler (2013)

Brightness affects mood and modulates emotion in the real world (Valdez & Mehrabian, 1994) and in movies (Chong, 2013; Tarvainen, Westman, & Oittinen, 2015). The brighter the image, the better viewers generally feel. Moreover, Keating (2011, p. 89) noted that some models of screenwriting recommend “that the writer place the lowest point of the emotional curve in the area around the ¾-point. This point can be called the darkest moment.” Is there a nonmetaphorical darkest moment along the movie’s emotional rollercoaster?

#### Methods

I analyzed the luminance in the larger sample of movies. To accommodate the sensitivity of the human eye, I converted the movies to grayscale, gamma-transformed (γ = 1/2.2 = .45) pixels within the eight-bit luminance range (0–255) in each frame, and averaged those values within each shot. I then normalized the 100 equal-duration luminance bins for each movie and took the medians across movies, as before. Results are shown in right panel of Fig. 3.

#### Results and discussion

A fourth-order polynomial fit is superimposed on the data, adjusted *R* ^2^ = .56, *F*(4, 95) = 32.5, *p* < .0001, and it is superior to lower order fits (adjusted *R* ^2^s = .46, .17, .13). Moreover in 10,000 two-fold cross-validation trials, the average training and test fits were again reasonably comparable (median adjusted *R* ^2^ = .43, and median *R* ^2 =^ .26; RMSDs = .136 and .162, respectively). And this time there were no main effects or interactions of release year or genres.

The quartic fit to the data shows a new and interesting pattern. Generally, movies are lighter over the course of the setup and the complication, they darken markedly across the development and then lighten again during the climax and through the epilog. Indeed, the difference in luminance between the complication and the development is striking, *t*(48) = 10.04, *p* < .0001, *d* = 2.90, and the linear rise throughout the climax is equally so, *t*(24) = 4.69, *p* < .0001, *d* = 1.91.

These results were a complete surprise. As Keating (2011) noted and as Bordwell (2008, p. 105) has elaborated, “most writers agree that the end of the second act”—here, the end of the development—“should be the ‘darkest moment,’ the point at which things seem to be utterly hopeless for the protagonist.” At least with regard to overall luminance, this appears to be literally true. Moreover, J. Smith (2015, p. 489) noted the function of this darkest moment: “By highlighting the enormous obstacles that the protagonist faces in achieving his or her goals, the ‘darkest moment’ provides maximal expressive contrast when the hero triumphs at film’s end.” Because the climax brightens markedly, this also provides the maximum luminance contrast. It appears that the emotionally darkest moment and its psychological functions have nonmetaphorical correlates in pixel measurements.

Continuing the emotions/luminance metaphor, if the ¾ point is the darkest moment, might the ¼ point (the lock-in) be the brightest? Since the data show that the entire setup and the beginning of the complication are bright, this is not quite so. Nonetheless, Lucey (1996) suggested the contrast; at the ¼ point the protagonist accepts the task of tackling the problem and things look good; at the ¾ point the protagonist looks defeated by the problem and things look very dark.

### Interim summary

By looking at shot durations and noncut densities, I found support for the narrative theory proposed by Thompson (1999) and Bordwell (2006). That is, (1) there is reasonable justification for the division of the narrative into four acts; (2) the critical data are generally inconsistent with Field (2005) and others in that they show that the complication is edited differently from the development in terms of shot durations and noncut transitions; (3) the climax shows considerable acceleration, represented by shorter duration shots and fewer noncuts, toward attainment of the final goal; and (4) within the climax there is strong physical evidence for a separate epilog. All attributes of this structure seem to have pervaded popular movies more or less uniformly for 75 years and more.

Motion and luminance also corroborate the four-act theme. Motion declines through the setup, stays roughly constant in the complication and development, rises sharply in the first part of the climax, and abruptly declines in the epilog. Luminance remains high throughout the first half of the setup, perhaps peaking at the beginning of the complication but declines rapidly through the end of the development, only to rise sharply through the climax.

There is also a nod toward something new. The initial downward swoop in both functions of Fig. 2 and in the left-hand panel of Fig. 3 appears to be too early in the narration (over the first 5 min or so) for an inciting incident. Moreover, this striking aspect in the data is at least as prominent as the changes of the epilog. Provisionally, with the idea of bookends that surround the *logos,* or conversational bulk of the movie, of the narration I call this early section the *prolog.*

One might reasonably assume that the extended duration of shots in the prolog would be due to being covered by credits, a film technique that began in 1960 in this sample. However, there are no differences in these results for Bins 1 through 5 in movies with trimmed versus overlaid credits. To be sure, I will search elsewhere for evidence for and against a prolog with and without credits, but I will also suggest that it, as Thompson (1999) has declared for the epilog, is an optional structure in a movie. Examples of movies with a “cold open”—that is, without a prolog or which have a deferred one for a later credit sequence—are *Erin Brockovich* (2000) and *The Social Network* (2010).

In sum, the data of the first four studies show striking articulation in movie narration. Most of these data are consistent with a four-act theory of popular movie narratives; they show that physical parameters of the surface form (of the syuzhet) in large sample of movies track the act structure (of the fabula) of the narrative. But not all aspects of movie narration do so. In Studies 5 and 6, I explore two dimensions that do not—one that seems structured more at the level of the whole movies rather than the act, and another that is structured at a smaller level—the scene.

## Part 2: Variations in film style that ignore Act structure

### Study 5: Seduction by nondiegetic music across the narration

The darkness, the strangers, the anticipation, the warm comfortable embrace of the cinema seat. We’re ready to experience some big emotions and the minute the music booms out, we are on board for the ride.

—Neil Brand (cited in Mackey, 2014)

My previous analyses have concentrated on the visual aspects of shots, but popular movies are obviously more than just visual narratives. Dialog aside, music has always accompanied movies, and indeed predates “the talkies.” For most of movie history the sound track has been bound into the analog film or digital format, but before that there was always musical accompaniment, often by live players. Kalinak (2010, p. xiii) suggested “film music guides our response to images and connects us to them” but how it does this seems logically odd. As Cohen (2001, p. 254) noted: “Music presumably adds to diegetic realism while providing nondiegetic, acoustical information that is completely incompatible with that realism.”

Nondiegetic (background) music has many functions in movies, but at least two are relevant here. First, even more than lighting, music sets a mood. It likely does this through associative processes that viewers share about the particular music: its key (major or minor), its genre (classical, popular, ethnic), its relative tempo, and its relative familiarity. Indeed, we have many physiological responses to movie music (Ellis & Simons, 2005), and as composer Neil Brand suggests in the epigram, it mentally envelops us in narrative, a phenomenon that Bruner (1991) called *narrative seduction* and Gerrig (1993) has called *narrative transportation*.

Second, music often provides advance information about an upcoming event or change in the plot, and indeed Boltz, Schulkind, and Kantra (1991); Magliano, Dijkstra, and Zwaan (1996); and S.-L. Tan, Spackman, and Bezdek (2007) have shown that movie viewers are sensitive to this fact. Filmmakers often use this foreshadowing so that viewers can be ready for what happens next, and this can greatly aid processing and memory. Thus, music puts the spectator in a state of attentiveness (Marshall & Cohen, 1988).

For these reasons, one can expect that filmmakers use nondiegetic music at points in the narration when important events and turns are about to happen. But does this too have a pattern across movies? Most of the music in the smaller sample was nondiegetic. That is, there were only a few moments of music played or sung “live” in the scenes of these movies. Cutting, Brunick, and Candan (2012) noted that the shots of action films are covered with music a bit less than 75% of the time, that those of comedies are covered about half the time, and that those of dramas a bit less than a quarter of the time. Moreover, they found no apparent trends over the period of movies investigated here, from 1940 to 2010. Thus, one should expect that the median use of music in the smaller sample of movies would be in a bit less than half the shots.

#### Methods

**Fig. 4:**
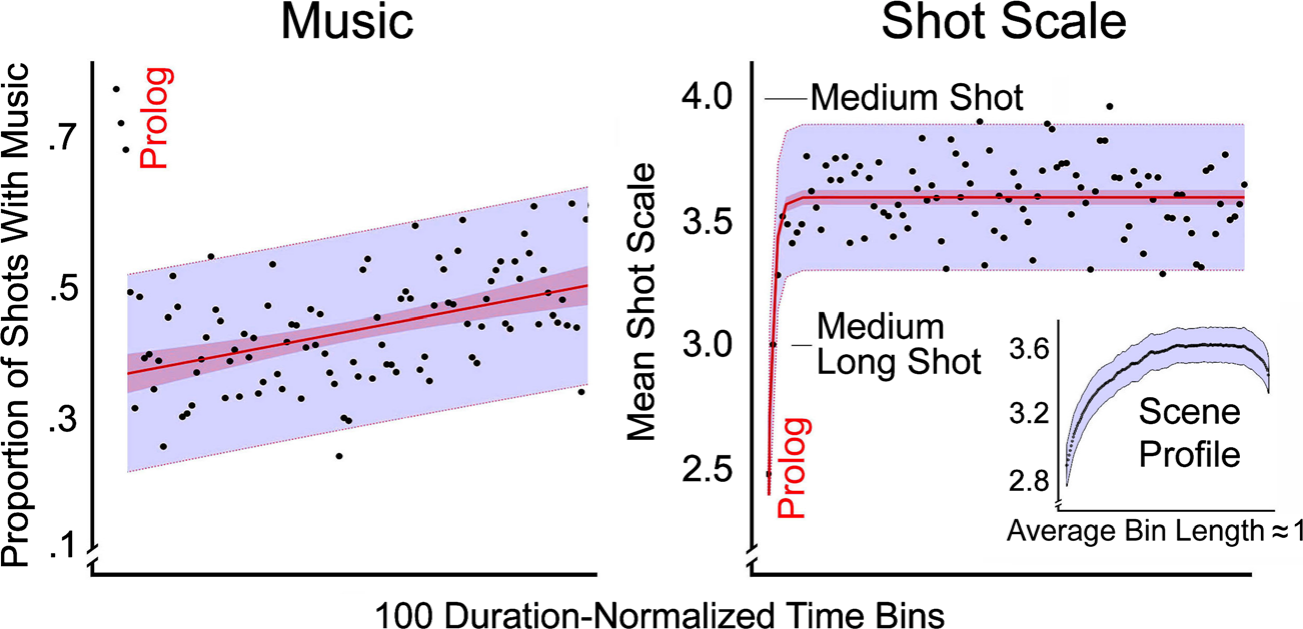
The distributions of music (Study 5) and of shot scale (Study 6) in 23 movies across the 100 duration-normalized time bins. Darker and lighter bands in both panels show 95% confidence intervals on the regression lines and the data, respectively. The insert in the lower right panel shows the duration-normalized profile of shot scales averaged across all scenes, with 95% confidence interval on the data (from Cutting, Brunick, & Candan, 2012)

As before, each movie was normalized into 100 equal duration bins. No other normalization took place. Instead, in each movie all shots associated with these bins were coded as to whether they had music covering them at any point (1 = yes, 0 = no), these values were then accumulated, averaged in each bin, and then aggregated across movies. Results are shown in the left panel of Fig. 4.

#### Results and discussion

The pattern for music as it is distributed across movies is different than for any of the previous variables. Basically, the early shots of a movie (the first three bins) are highly likely to be covered with music (nearly 75%), but the probability then plummets to about 35%. One might think that there would be a difference here between movies with trimmed versus overlaid credits, and there is but in a surprising direction. Those movies that had their credits trimmed had more nondiegetic music in the first three bins than those with overlaid screen credits (87% vs. 65%), before plummeting to values below those with overlaid-credits (33% vs. 44%) for Bins 4–10. Because the seven movies in the former group are all older movies, this may reflect stylistic differences that have changed with time.

Over the remaining bulk of the movie, the music gradually and linearly increases to accompany over half of all shots. I fit these later data with a linear regression so show its sharp contrast with the prolog. The rising change is marked, *R* ^2^ = .21, *F*(1, 96) = 26.3, *p* < .0001, and with no articulation within it, that might suggest different uses of music within different acts.

In other words, after an initial flurry of music at the beginning of the setup—again, more strong evidence for the existence of a prolog—filmmakers drop back and gradually build up the nondiegetic music as the narration progresses. Likely they do this in part to increase tension and further induce viewers to remain transported by the narrative. This more-or-less uniform trend would also suggest that music is bound to the fabula of the whole movie, not to that of the act structure of narrative. Nonetheless, it also likely reflects the emotional content in separate scenes scattered throughout the different narratives, and that the scenes get linearly more intense across the narration. I will suggest later in the discussion of Study 6 that the organization of individual scenes is also quite independent of the four acts, and that averaging across scenes creates no special pattern in the act structure of movies.

### Study 6: Sizing Up characters in visual narration

The close-up has inspired fascination, love, horror, empathy, pain, unease. It has been seen as the vehicle of the star, the privileged receptacle of affect, of passion, the guarantee of the cinema's status as a universal language.

—Mary Ann Doane (2003, p. 90)

The use of different shot scales—a close-up, a long shot, or something in between—is a staging decision within film style. The director, cinematographer, and editor choose how much of the frame should be filled by a character’s face and body. Cutting (2015) found that 90% of all shots in the smaller sample have the face of at least one character in them, and the remainder are usually inserts (shots containing objects that the viewer needs to know about or that lend tone to a scene—such as a black cat in a dark alley), point-of-view shots (objects that a character has looked at in the previous or sometimes subsequent shot), or establishing shots (long shots of landscapes or cityscapes). Obviously, the larger the character in the frame, the easier it is to judge the response of that character to the events in the narrative (Cutting & Armstrong, 2016). The motivating question then is: Is there variation in shot scales across the larger units of the narrative?

#### Methods and results

Using the shot scale data of Cutting, Brunick, and Candan (2012), I divided the movies in the smaller sample into 100 bins, determined the median shot scale within each bin for each movie, and then averaged these data across movies. Unlike in other studies, I did not normalize the data, in part to compare them with previous work.

Results are shown in the right panel of Fig. 4 and fit with an inverted negative exponential, adjusted *R* ^2^ = .51, *t*(96) = 10.15, *p* < .0001, *d* = 2.07. Notice that except for the first three bins there is no patterned change in the median shot scale across the rest of the movies, and even this inflection is surely because every movie must have a beginning of a first scene, which starts with Bin 1. Thus, whatever information about scene change is carried by shot scale, filmmakers use none of it to mark the changes across acts. Furthermore, the long flat section of the scale values again implies an independence of film form for acts and scenes.

#### Discussion

With respect to any theory of the larger narrative, the focus of this article, shot scale is essentially unvarying. However, at a more local level—the level of the scenes within the narrative—the pattern is much different. On average the movies in this sample have a new scene or subscene every 55 seconds. Cutting, Brunick, and Candan (2012) length-normalized all of these scenes (~3,200) and then calculated the average profile in terms of shot scale. The insert in the right panel of Fig. 4 shows these data—an arc that might have pleased Aristotle, having a beginning, a middle, and an end. Scenes tend to begin with a longer-scale shot and become shorter as the camera moves in to show more of the faces of the characters, and with a lengthening at the end of the scene when the cinematography often backs off before a cut to a new scene. Cutting, Brunick, and Candan (2012) found that shot scale is the most important cue for scene change, followed by transition type (cuts vs. noncuts), shot duration, color, and, much less prominently, luminance and motion. That the latter two provide patterned information about act structure suggests that different physical parameters of movies are associated with different-sized units of narrative structure.

### Interim summary

Music and shot scale offer no additional information about the act structure of movies. The use of music appears to be conditioned on the stylistic decisions of the filmmakers at the level of whole-movie narration, building incrementally throughout, whereas the use of shot scale is a function of stylistic decisions and the level of the scene, which then average out as the succession of scenes proceeds at different rates in different movies. The general independence of these levels, I would contend, adds flexibility to narration. Among the three proposed here, one source of information tugs at the attention of the viewer in the domain of 2 hours or so (music), others in the domains of about a half-hour or less (shot duration, shot transitions, motion, and luminance), and still another in the domain of a minute or so (shot scale).

Moreover, both music and shot scale provide continuing support for the prolog—the amount of nondiegetic music is greater, the shot scale is longer, and, looking back to Fig. 2, the shot durations are longer and the transitions between those shots are more likely to be dissolves. The evidence is sufficiently strong for a prolog within the setup that I have added it to Fig. 1 as an elaboration of Thompson’s (1999) scheme.

The previous six studies have dealt with narrational information from the shots of movies—durations, transitions, motion, luminance, music, and shot scale. The next four deal with aspects closer to the narrative proper—determining when characters are introduced, when characters talk to one another, when genres might diverge, and when scenes change.

## Part 3: Functional variations in narrative structure across acts

### Study 7: Character introduction and the setup

In all the manga that I have read and anime I have watched, the protagonist always appears in the first chapter of manga or first episode of an anime.

—Toshinou Kyouko (2014)

The defining characteristic of the setup is the introduction of major characters, but when exactly do characters first appear? Cutting and Iricinschi (2015) noted that the smaller sample of movies investigated here have an average of 10 characters that appear in at least 8% of all scenes in a movie. I will call most of these the *other characters*, plus add a few that are important to the plot of a few movies. From these I single out the protagonist or protagonists (one to three).

#### Methods

**Fig. 5:**
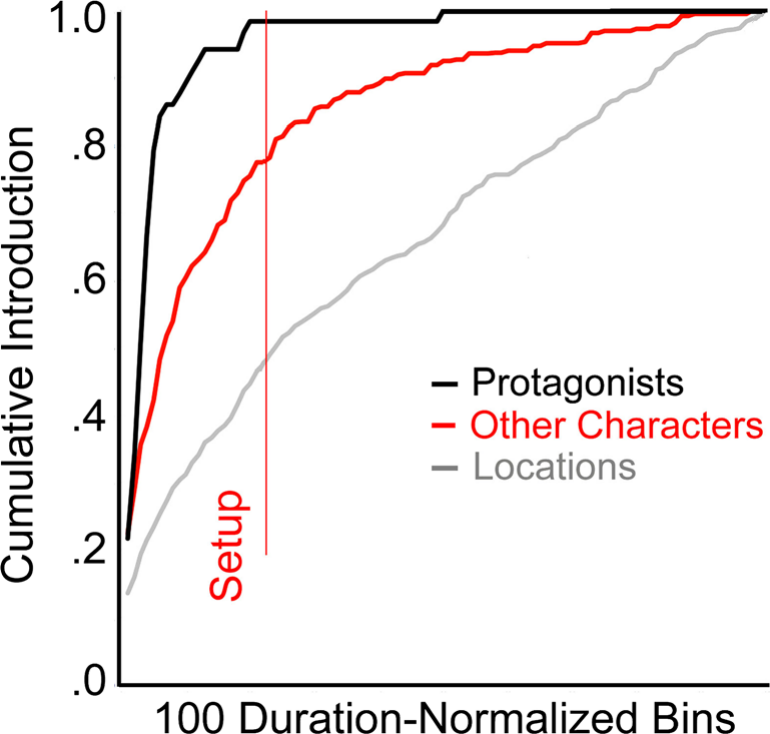
Cumulative records across the smaller sample of 23 movies concerning the introduction of the protagonists and the other main characters appearing in at least 10% of the scenes, compared with the cumulative record of the appearance of new locations (from Study 7)

In the same way I have divided these movies before, I noted when each character first appeared and assigned that appearance to the appropriate bin. I normalized the 100 bin entries so that together they summed to 1.0. Thus, in a movie with 10 characters, the appearance of each counted 0.1; and for one with only five characters, each would count 0.2. Lead character were done in the same way. For a movie with three protagonists, each would count .33; and for one with two, each would count .50. Again, each movie contributed equally to the results. For comparison, I plotted the appearance of new locations in each film as well, and assigned them to the appropriate bins. The average movie in this sample had 31 locations, so weights per bin were adjusted accordingly. Unlike other analyses, however, I accumulate the results until they added to 1.0, and the results are shown in Fig. 5.

#### Results and discussion

Nearly 98% of all protagonists are introduced within the time frame of the setup as I have defined it, following Thompson (1999). Moreover, those few who had not been introduced were among the movies with more than one protagonist. Thus, all movies had at least one introduced during the setup, and 80% of all main characters are introduced in the setup. Such early appearance is not true for locations. Just under half of the locations have been seen during the setup, and these are then introduced fairly uniformly throughout the rest of the movie. Notice also that half of the main characters and 90% of the protagonists have been introduced in the first three bins.

Thus, and again, there is a heavy emphasis on a difference in the structure of the movie within the first 5 to 7 minutes; the prolog typically introduces the protagonist(s). Notice that there are often 10 to 20 minutes remaining in the setup after the introduction of a protagonist. Clearly, this is when backstory and exposition occur as well as the introduction of subsidiary characters. Following Gernsbacher (1995), this is when the foundation of the narrative is laid. Finally, notice that the setup is not strongly identified with the introduction of locations. Character introduction seems logically necessary in the setup, but what’s going on in subsequent acts?

### Study 8: Conversations in the complication and development

Despite critics’ complaints that movies are packed with chases, explosions, and gun battles, the standard scene remains a conversation.

—David Bordwell (2005, p. 22)

Music is not the only important content in the audio track; indeed, speech is even more dominant. Cutting and Candan (2015) categorized every shot in the smaller sample of movies into 15 different types of shots. Five of these concern conversations: master shots, which show all conversants within the frame; and shot/reverse shots, which come in four varieties, typically with alternating camera angles. The most common is the shot/reverse-shot, where the camera has only one talker in frame at a given time but alternates characters across shots. A variant of this is the over-the-shoulder shot, where the camera is behind one character whose back is turned and is focused on the other character, who is talking. A third can be either over-the-shoulder or without the turned-away character but with the character facing the camera not talking, but listening to the other. This is called a reaction shot. Finally, there are occasional conversational shots over telephones, intercoms, or holographic devices in science fiction movies that are staged in a similar way to regular conversations. In addition, some shot/reverse-shot sequences have more than two characters, but these are typically handled in the same way. In the smaller sample, as implied by Bordwell (2005) in the epigram, these five types dominate movies; they occur in 72% of all shots in dramas, in 60% of all shots in comedies, and in 43% of all shots in action films.

#### Methods, results, and discussion

**Fig. 6:**
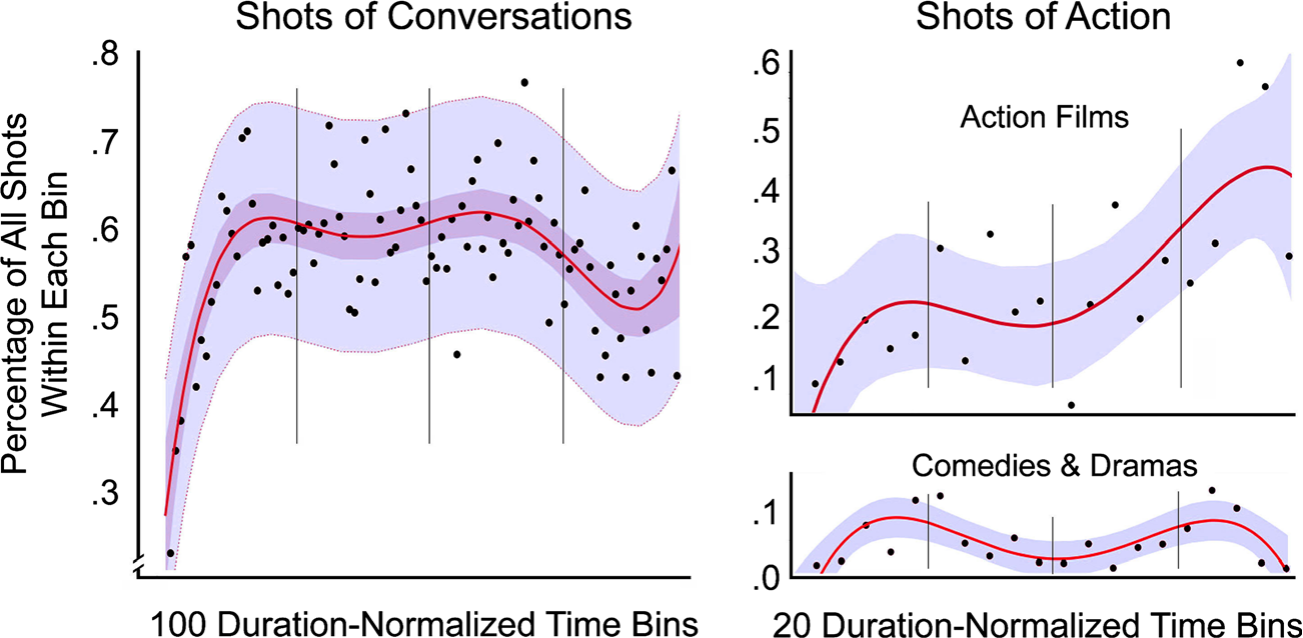
Median frequencies of conversation shots (master shots, over-the-shoulder shots, shot/reverse shots, reaction shots, and telephone shots) across 100 duration-normalized bins (Study 8) and of action shots across 20 bins (Study 9) across the narratives in 17 movies, 7 action films and 10 dramas and comedies. The dark purple band in the left panel shows the 95% confidence intervals on the regression line, and the lighter purple that for the data. The lighter purple bands in the right panel are the 95% confidence intervals on the regression lines. Vertical lines separate the setup, complication, development, and climax as in Figs. 2 and 3

All conversational shots were apportioned to the 100 bins according to the lenght of the movie, and coded for the presence (1) or absence (0). These were then averaged within bins and the mean proportion per bin is shown in the left panel of Fig. 6. A fifth-order polynomial is fit to the data, adjusted *R* ^2^ = .40, *F*(5, 94) = 14.14, *p* < .0001, and it is superior to all lower order fits (adjusted *R* ^2^s = .30, .29, .24, .00). The results of 10,000 two-fold cross-validation assessments resulted in similar fits for training and test sets (adjusted median *R* ^2^ = .34, and median *R* ^2^ = .31, and RMSDs of .366 and .404, respectively).

Notice three trends in the data. First, conversations are few in the prolog and their level doesn’t stabilize until near the end of the setup. Moreover and again, there is no difference between movies with and without covering credit sequences. Second, the conversational density is quite constant but noisily varied through the complication and development. Third, conversations decline during the initial portion of the climax, but rise again in the epilog. Thus, for a bit more than half of a movie—from the end of the setup, through the complication and development—has a fairly constant density of conversations, occupying about 60% of all shots. The decline in conversations at the beginning of the climax suggests that it is finally time for the protagonist(s) to act, and not to talk. But again, the most striking trend is in the prolog, where few conversations occur and gradually increase over the first two-thirds of the setup.

Shots of conversations are the most common shots in filmmakers’ armamentarium, and these are most prominent in the complication and the development. But other shots may be more distinctive. As suggested by Bordwell in the epigram, perhaps the most salient—and the type that most clearly separates genres—is the action shot. If one is looking for a general formula for movies, surely one of the best ways to test this idea is to look for a shot type that distinguishes genres and may rupture the idea of a uniform movie format.

### Study 9: Action shots in the climax and elsewhere

. . . swordsman and swordswoman confront enemy warriors in a bamboo grove. It is no ordinary combat. The fighters leap twenty feet in the air, pivoting and somersaulting, sometimes clashing with one another.

—David Bordwell (2000, pp. 1–2)

Movies, like life, are basically about talking and doing. Most “doing” in movies entails a moving character or a moving object (typically a vehicle). However, some “doings” are well beyond the norm, and these are captured in action shots. In their taxonomy of shot types Cutting and Candan (2015) defined an action shot as one with beyond-normal physical activity—not only those in fights of all kinds but also those in sports, accidents, explosions, chases, building collapses, and other more or less extreme events. Helpfully for classification, in sequences these shots are almost always covered by nondiegetic music.

Not all shots in action films are action shots, but many are. Moreover, and equally important, a number of action shots are found in films that are not action movies. Cutting and Candan found that action shots comprise an average of 30% of all shots in the action films of this sample. Unsurprisingly, action shots in dramas are rare (1.7%). Indeed, there were none in three of the eight dramas in the smaller sample. Nonetheless, action shots in comedies are more prevalent (6.4%), and only one comedy of the eight failed to have an action shot. Thus, it is worth comparing action shots in action movies versus those in the other films.

#### Methods, results, and discussion

Because action shots are relatively sparse in most of these movies, I divided the movies into 20 time bins, found the proportion of action shots within each bin, and then averaged those values across movies. The data for the seven action films and the 10 remaining comedies and dramas separately are shown in the right panel of Fig. 6. There is a clear main effect separating the two genre groups,(*t*(36) = 7.32, *p* < .0001, *d* = 2.44, and an interaction as well, *t*(36) = 3.43, *p* = .0015, *d* = 1.14, with action shots increasing across the length of action films.

The data for the nonaction films are best fit by a fourth-order polynomial, adjusted *R* ^2^ = .38, *F*(4, 15) = 3.91, *p* = .023, which is superior to lower order fits, adjusted *R* ^2^s = .00, .00, .00). Notice the two humps, one at the boundary between the setup and the complication and the other just into the climax. The latter makes considerable sense—there should be a rise to higher activity generating more action shots, in the climax, and these should fall off during the epilog. The first hump, on the other hand, was a surprise. Action shots are just as prevalent here as in the later cluster and suggest that part of the inciting incident or the lock-in at the boundary between the setup and complication in may often be preceded and followed by strenuous activity.

Although the data are noisy, again the action films show a similar pattern but with greater frequency and an interaction showing increasing frequency across the runtime of the movie. The fourth-order polynomial fits reasonably well, adjusted *R* ^2^ = .42, *F*(4, 15) = 4.42, *p* = .015, but it is not different from the lower-order polynomials (adjusted *R* ^2^s = .38, .40) or the linear fit (adjusted *R* ^2^ = .40). One possible reason for the relative weakness of the higher order polynomial fit was mentioned earlier in the context of Study 3— action sequences can occur at almost any point in the runtime of an action movie, and the relative noise in these data bear this point out. Another is that there are only seven action movies sampled.

Nonetheless, I find the general parallel between the fourth-order fits to the action and nonaction films worth preserving, suggesting that all genres have more or less the same structure with the pattern in action films considerably ramped up. The uptick in action shots at the boundary between the setup and the complication also serves to further distinguish those two acts, and given some substance to the notion of a lock-in, the turning point between the two acts.

If character introduction and laying the foundation of the narrative is the function of the setup, and if conversational exchange is the function of the complication and the development, action is clearly the function of the first part of the climax. How then do these facts affect the scene structure of the narrative?

### Study 10: The pacing of scenes and narrative shifts

In drama, *scene* refers to a division within an act of a play, indicated by a change of locale, abrupt shift in time, or the entrance or exit of a major character.

—Kirk Polking (1990, p. 405)

Let me now shift to discussion of the scene. Continuity and discontinuity, the psychological impression in movies of ongoingness versus change, are not what they might first appear. Strikingly, despite the glaring and abrupt physical differences, cuts do not always disrupt the continuity of the unfolding story (see, e.g., Zacks, 2015). But discontinuities do occur, and they occur with cuts and other transitions at scene boundaries, at what can be called narrative shifts (see Zwaan, Langston, & Graesser, 1995; Zwaan, Magliano, & Graeser, 1995). Narrative shifts can occur in a number of different ways, but for movies I will confine my discussion to three dimensions as suggested by Polking in the epigram—changes in *location*, changes of *characters* as they arrive and leave, and changes of *time* such as in an ellipsis, a flashback, or a dream (see Cutting, 2014; Cutting & Iricinschi, 2015; Magliano & Zacks, 2011; Zacks, 2015).

By this formulation there are seven kinds of narrative shifts generated by the presence or absence of a shift along the three dimensions. Shifts and nonshifts at shot transitions yield 2^3^ or eight possibilities, but when all three dimensions do not shift the result is continuity. This fact subtracts one out, yielding seven. Shifts in location, regardless of whether or not there is a shift in one of the other dimensions, are quite common, occurring in the smaller sample an average of 98 times per movie. Shifts in characters are a bit more prevalent, averaging 107 per movie, but shifts in time are less so, averaging only 49 per movie. Again, typically more than one dimension shifts at a time so the average number of narrative shifts of one kind or another in this sample of movies is 128 (Cutting, 2014). How are narrative shifts distributed across the length of movies?

#### Methods

Cutting, Brunick, and Candan (2012) had viewers segment 24 movies into scenes, three viewers per film. Each viewer marked the frame number where a new scene began. They agreed 91% of the time (mean κ = .56), and we analyzed every scene boundary that each viewer marked. Cutting and Iricinschi (2015) later marked the number of narrative shifts that did and did not correspond within segmentations in each movie and found that viewers had strongly endorsed these data (*d*’ = 2.38; Cutting, 2014).

Analyses below are carried out on the three types of narrative shifts—location shifts whether or not characters or time changed, character shifts whether or not location and time changed, and time shifts whether or not locations and characters changed. As I did above for noncuts and because these data are sparser than those of other studies, I divided the 23 movies in the smaller sample into 20 equal length time bins, recorded the number of narrative shifts in each, and normalized them to sum to 1.0.

#### Results and discussion

**Fig. 7:**
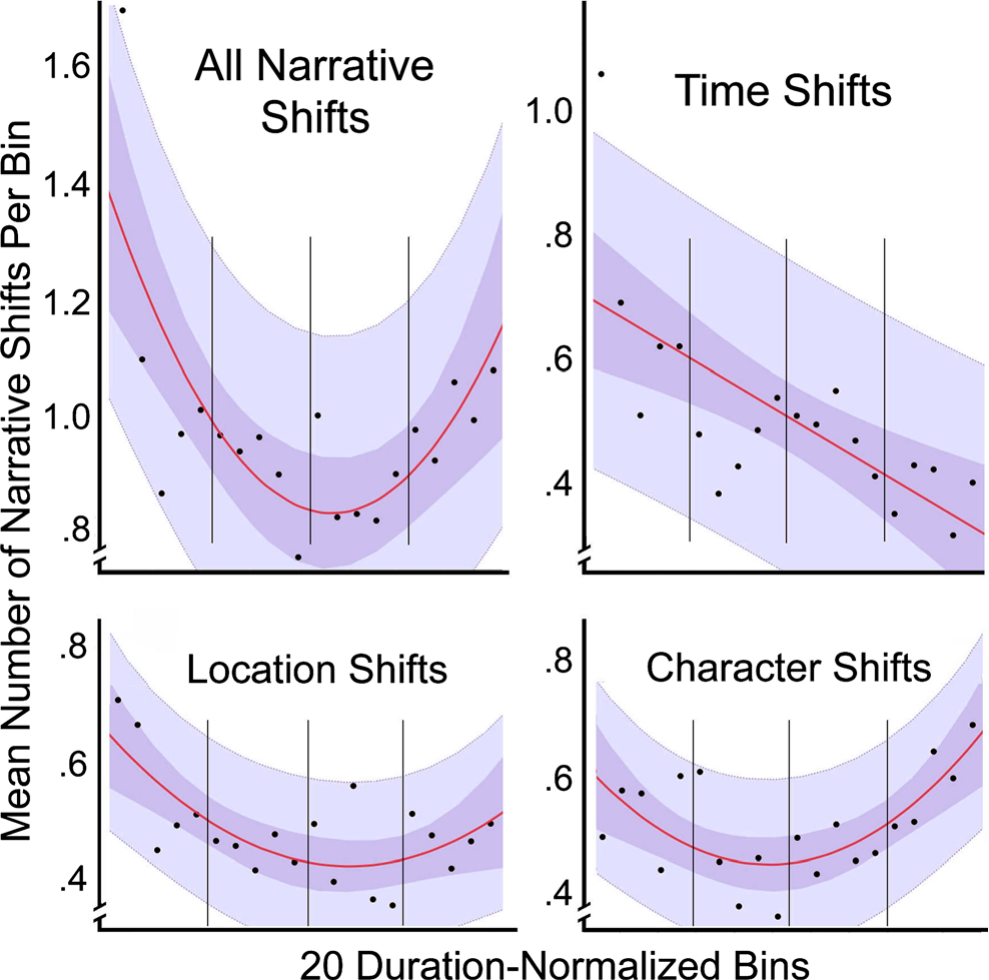
Normalized density distributions across 20 consecutive, duration-normalized bins in 23 movies, for all narrative shifts, and all changes in location, in characters, and in time (Study 10). Dark and light purple bands show 95% confidence intervals on the regression lines and data, respectively. Vertical lines separate the setup, complication, development, and climax as in Figs. 2 and 3

Narrative shifts of all three types are considered together in the upper left panel of Fig. 7, plotted as the average number in each of the 20 consecutive bins. A quadratic function fits these data quite well, adjusted *R* ^2^ = .50, *F*(2, 17) = 16.7, *p* = .0008, with the main pairwise result across acts occurring as a marginal difference between the development and the climax, 0.90 versus 1.01, *t*(8) = 2.49, *p* = .037, *d* = .37. Given that shorter shot durations and more motion occur during the climax this should not be a surprise. Note that again the first bin (the first 5 to 7 min of a movie) is an outlier, with many more scene or subscene changes than at any other point in the movie. And again, this is evidence for a separate prolog within the setup. This is likely because the typical movie often dodges around to different locations with different characters in an exposition phase of the narrative.

The general shape of the function promotes an implication. Given that there are fewer narrative shifts in the middle of a movie, those scenes must be longer than elsewhere. Indeed, the length of scenes and subscenes in the setup and climax averages 47.1 s, but that for scenes and subscenes in the complication and development averages 60.1 s, *t*(98) = 89.6, *p* < .0001, *d* = 18.1. This trend seems allied with the results of Study 8, which showed that the middle of a movie has many more conversations than at either end. Conversational scenes are longer.

As shown in the lower panels of Fig. 7 the shifts in location and characters are also both best fit by a similar second-order polynomial, adjusted *R* ^2^s = .440 and .437, *F*s(2, 17) = 9.42 and 9.24, *p*s < .01, respectively, with bins at either end of the movie, the setup and the climax, with a few more shifts. Since these two types of shifts are the most common and most correlated it is not a surprise that the summary shift pattern in the upper left panel is essentially the same. However, none of the pairwise comparisons across the acts for location shifts were statistically reliable, and among the character shifts only the development to climax difference was marked, *t*(8) = 2.58, *p* = .032, *d* = 1.82. The data and quadratic fit for locations are a bit more lopsided than that for characters, with location changes more frontloaded in the narrative.

As shown in the upper right panel of Fig. 7, the plot for narrative shifts in time is different in shape than for locations and characters, combined for this analysis, *t*(36) = 3.33, *p* = .002, *d* = 1.11, with a linear fit to the data best, adjusted *R* ^2^ = .46, *t*(18) = -4.12, *p* = .0006, *d* = 1.94. The differences in time shifts from the setup to complication, *t*(8) = 2.42, *p* = .041, *d* = 1.71, and from the development to climax, *t*(8) = 3.39, *p* = .009, *d* = 2.39, were marked. In other words, time shifts become fewer across the narration. This result makes sense as the protagonist moves toward achieving her goal. Diegetic time becomes more like real time.

It is interesting to consider two types of time as discussed in the narratology of literature, and how these become particularly acute in movies. This comparison concerns *narrative speed* (see, e.g., Genette, 1980; Hume, 2005). Paxson (1994, p. 37), referring to Genette’s scheme, outlined four relations between narration time (the time it takes to tell that part of the story) and narrated time (the time passing in that part of the story, or diegetic time): “Narrative speed divides into four ‘tempos’: ellipsis, summary, scene, and pause. ‘Scene’ is the tempo in which narrated time is equivalent to the time of narration. ‘Ellipsis’ and ‘summary’ are tempos in which narrated time is greater than time of narration; ‘pause’ is a tempo in which narrated time is less than narration time.”

As Paxson and Genette define them summaries in movies are rare. They typically occur in voiceovers often in the prolog (as in *All About Eve,* 1950) or in text-overs often in the epilog (as in *Erin Brockovich,* 2000, and *The Social Network,* 2010). Pauses are also rare but can occur when the cinematography freezes frame while a narrator continues, as happens in *All About Eve* (1950) and *Goodfellas* (1990). On the other hand, the tempos of ellipsis and scene are the backbones of movie narration (although Genette’s account of ellipsis seems not to include flashbacks).

The data of Fig. 7 show that narrative and narration speed converge over the course of movies in this sample, likely because there are fewer ellipses. This undoubtedly gives the viewer a sense of urgency as the narrative increasingly proceeds in real time as it moves toward and into the climax. The reason for the trend in the data may stem from a strong organizing principle in narratives of all movie genres—the deadline (Bordwell & Thompson, 2011). That is, a goal is set and must be attained by the protagonist at a particular time. Movie narratives, as they approach that deadline, appear to have a strong tendency to proceed with the coupling of diegetic (narrative time) and real time (narration time). Action films particularly have this feature (e.g., *Mission: Impossible II,* 2000; *Inception,* 2010), although it occurs in comedies (*Philadelphia Story,* 1940) and dramas (*Witness,* 1985) as well.

### Interim summary

Study 7 affirmed the functionality of the setup. Most characters, and particularly the protagonist(s), are introduced and their goals become known. As Gernsbacher (1995) would note, this establishes a foundation on which the relations among subsequent events can be laid. Study 8 continues the thread of functionality, showing that the setup is relatively devoid of conversations but that the complication and development are rife with them. Clearly, these conversations carry the bulk of the narrative progression and conflict within the plot. Study 8 also showed that conversations become less prevalent at the beginning of the climax, when the protagonist is driven to action, but their frequency returns for the epilog.

Study 9 follows the most physical activity in the narration, finding in dramas and comedies that it peaks at the end of the setup and again at the beginning of the climax—both times when conversations are less. It also showed this pattern for action films but with an additional underlying increase throughout.

And Study 10 plots the frequency of scenes and the changes between them. Scenes are longer in the complication and development, likely because of the conversations, and shorter in the setup and climax. Location and character changes across scenes follow this pattern, but time changes do not. Instead, time changes become incrementally less frequent. That is, narration time and narrative time converge, likely because of the complications of story structure around a deadline that must be met.

## Part 4: Dimensionalizing movie narration

The previous 10 studies have each taken single attributes movie narration and explored how those unfold over the course of the average film. Their results provided useful information, much of which validated a four-act narrative theory of popular film. However, it seems logically implausible that these results would reflect ten independent dimensions of movies. Instead, since the structure of the world around us is a high-dimensional but vastly intercorrelated space (Edelman, 2008), movie structure should be as well. Thus, the last two studies seek to place these attributes in a lower dimensional framework.

### Study 11: From ten variables to three principle components with five correlated dimensions of film style

Simply put, cognitivists like film. We are interested in understanding the basic processes of how movies work: how they are structured, how they convey meaning and evoke emotion.

—Greg M. Smith (2014, p. 286)

In the previous 10 studies I analyzed movies in 10 different ways. Some connections became obvious, as in Studies 1 and 2, with the strong negative correlation between transitions of all kinds and noncut transitions. Other oppositions, however, are less obvious. One way to explore these is through principal component analysis. This statistical technique converts different sets of matched data into a set of linearly uncorrelated variables called principal components. Given 10 inputs, one could search for as many as 10 components, but this would be unhelpful. Instead, I will search for a reasonable solution with as few components as seems warranted, and then search for dimensions of film style within them.

#### Methods and results

The median bin values from each study were entered as columns in a larger data set. I used the raw medians for the 100 bins from most studies (1, 3, 4, 5, 6, and 7), and I interpolated values to achieve 100 estimates from those studies with only 20 bin medians (2, 9, and 10).^11^ That is, for example, what were Bins 1 and 2 became Bins 1 and 6, with intermediate bins having values linearly interpolated in between. This expansion procedure was continued (Bins 2 and 3 became Bins 6 and 11; 4 and 5 became 16 and 21, etc.) throughout until there were 100 bins. Since there can be range effects in principal component analysis, all values from each study were first normalized (mean = 0, standard deviation = 1). With the 10 normalized inputs I then ran the analysis. Seven components accounted for reliable variance, but the first two components were by far the most potent (χ^2^s > 350, *df*s < 46, *p*s < .0001), with the first accounting for 43.4% of the variance and the second 20.7%.

**Fig. 8:**
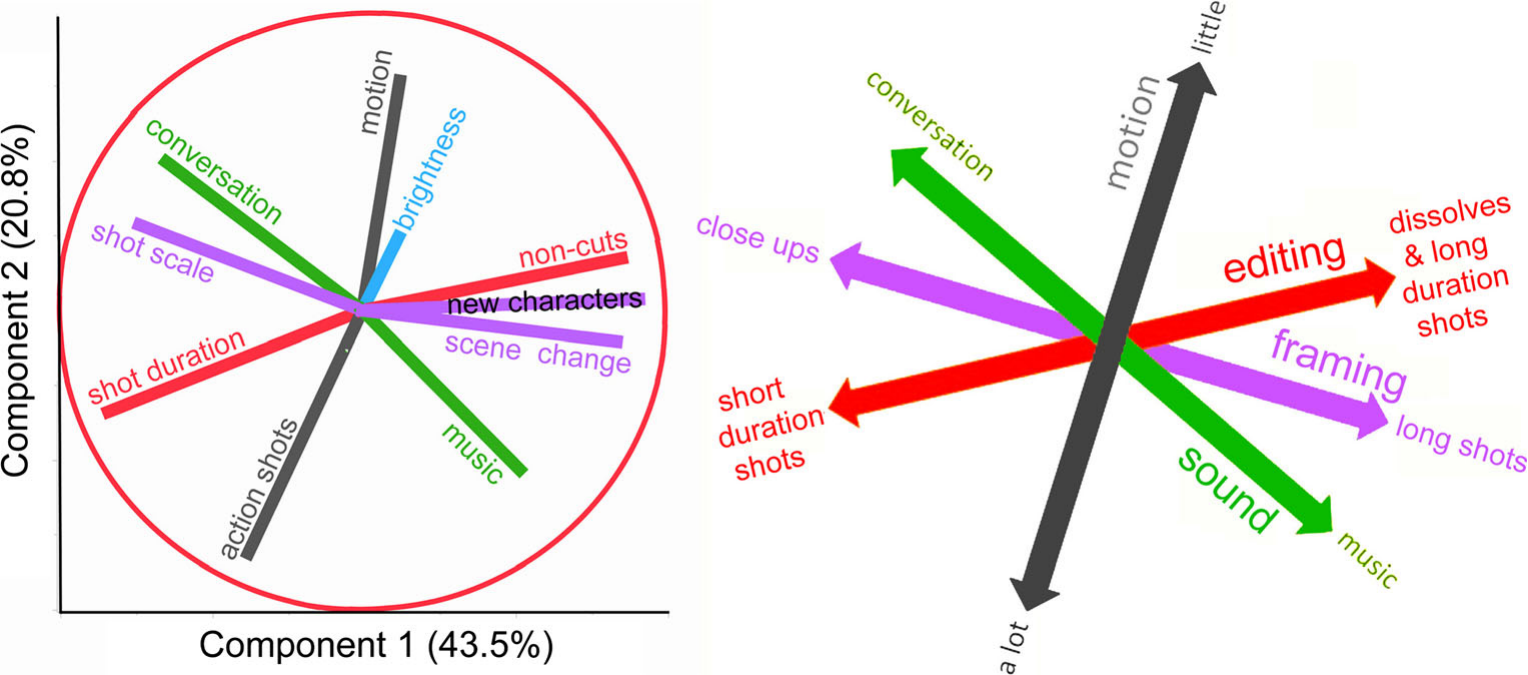
The left panel shows a loading plot of the first two principal components and the vector strengths of the data in those components for the variables from each of the 10 preceding studies. These components account for about 65% of the variance in the data. The right panel (leaving out brightness) provides a dimensional interpretation of these data as four correlated, opponent continua

These results can be most easily visualized in a loading plot of the two components. Following Pythagoras, the maximum variance accounted for across two components is the square root of the sum of the squares of component correlations for each variable. Thus, the results can handily be plotted in a circle. Such a loading plot of the 10 variables is shown in the left panel of Fig. 8, with the first component forming the horizontal axis and the second forming the vertical. Notice that all but one of the data vectors, brightness from Study 4, approaches the circular limit. The average length of the nine longest vectors is .84. This means that the data of nine of the 10 studies is well captured by these two components. Inspections of other loading plots revealed that brightness is the only variable well represented in the third component (load *r* = .90), which accounted for an additional 11.6% of the variance.

As expected the vectors for Studies 1 and 2, transition densities and noncut densities, are opposed and can be combined, as shown in red in the right panel of Fig. 8. This result is because their data were so strongly and negatively correlated (in the linearized plot, *r* = -.75). But notice that other oppositions are nearly as prominent. For example, the vectors for the data of conversations (Study 8) and of music (Study 5), shown in green, are also opposed, *r* = -.48, *t*(98) = -6.3, *p* < .0001, *d* = 1.09. This makes sense given that diegetic music is rarely heard in the background of conversations and that nondiegetic music often occurs when no character is talking.

Another set of oppositions, shown in purple, concerns the data for shot scale (Study 6) and those for two other vectors – scene change (Study 10) and the introduction of new characters (Study 7), with the latter two highly correlated, *r* = .83, *t*(18) = 7.48, *p* < .0001, *d* = 3.52. This too makes sense. That is, shorter scales—close-ups and medium shots—are used in the middle of scenes, whereas longer scale shots tend to be used at the beginnings of scenes, the time when new characters are typically introduced.

Finally, motion and action shots, shown in black, are closely related, *r* = .50, *t*(18) = 5.72, *p* = .0001, *d* = 1.15, and are generally independent of these other vectors. Luminance, shown in light blue in the left panel, is ill fitting in this plot.

#### Discussion and further analysis

These patterns allow for a set of interpretations, shown in the right panel, of four correlated bidirectional dimensions in the data. This type of representation generated from a loading plot is nonstandard, so let me work through it. The dimension whose component vectors fit best within the loading plot is shown in red, and it is strongly related to the first component. It concerns short duration shots versus long duration shots, and the latter’s alliance with dissolves as transitions. I will call this the *editing* dimension.

The second best fitting dimension, is in black, and is most closely aligned with the second component. It combines motion and action shots, and I will call it the *motion* dimension. The third dimension, in green, pools music and conversations. I will call it the *sound* dimension, and it cuts diagonally across the two components. The fourth dimension concerns *framing* and combines three vectors. It pits the values of shot scales with character introduction and scene changes. This dimension is also closely aligned with the first component. And the one dimension not shown and strongly represented in a third component is *lighting*.

What is particularly pleasing about this arrangement are the cross-dimensional correlations that they suggest, replicating many findings elsewhere. The first comparison is the strong correlation between shot duration and shot scaling, *r* = .78, *t*(98) = 12.2, *d* = 2.46, with longer-scaled shots being associated with longer duration shots (and scene changes and character introduction) and shorter scaled shots (medium shots and close-ups in midscene) associated with shorter duration shots. The association between shot scaling and duration was broached by Bordwell (2006) and explored and supported in detail by Cutting (2015) and Cutting and Armstrong (2016).

Moving counterclockwise, a second dimensional association is that between shot scale and sound, *r* = .55, *t*(98) = 6.45, *d* = 1.30. This is valid because conversations are typically filmed with medium close-ups and without music. Finally, there is an association between shot duration and motion (*r* = .46, *t* = 5.13, *d* = 1.04), with shorter duration shots on average having proportionately more motion across frames than longer durations shots, a result supported by the analyses of Cutting, DeLong, Brunick, Iricinschi, and Candan (2011b).

In this manner, a principal component analysis allowed the derivation of four correlated conceptual dimensions of movie narration. These are laid out across two components and shown in the left panel of Fig. 8. Moreover, these dimensions seem to capture well the relations among nine of the physical dimensions explored in the studies presented here. So far, of course, this analysis ignores the time course of the narration, but more can be done within this componential framework. In particular, the dimensional analysis of Fig. 8 can be explored within bins appropriate to the sections of movies.

### Study 12: Six snapshots of movie narration

Narration is more than an armory of devices; it becomes our access, moment by moment, to the unfolding story. . . . Narration in any medium can usefully be thought of as governing our trajectory through the narrative.

—David Bordwell (2008, p. 12)

#### Methods

**Fig. 9:**
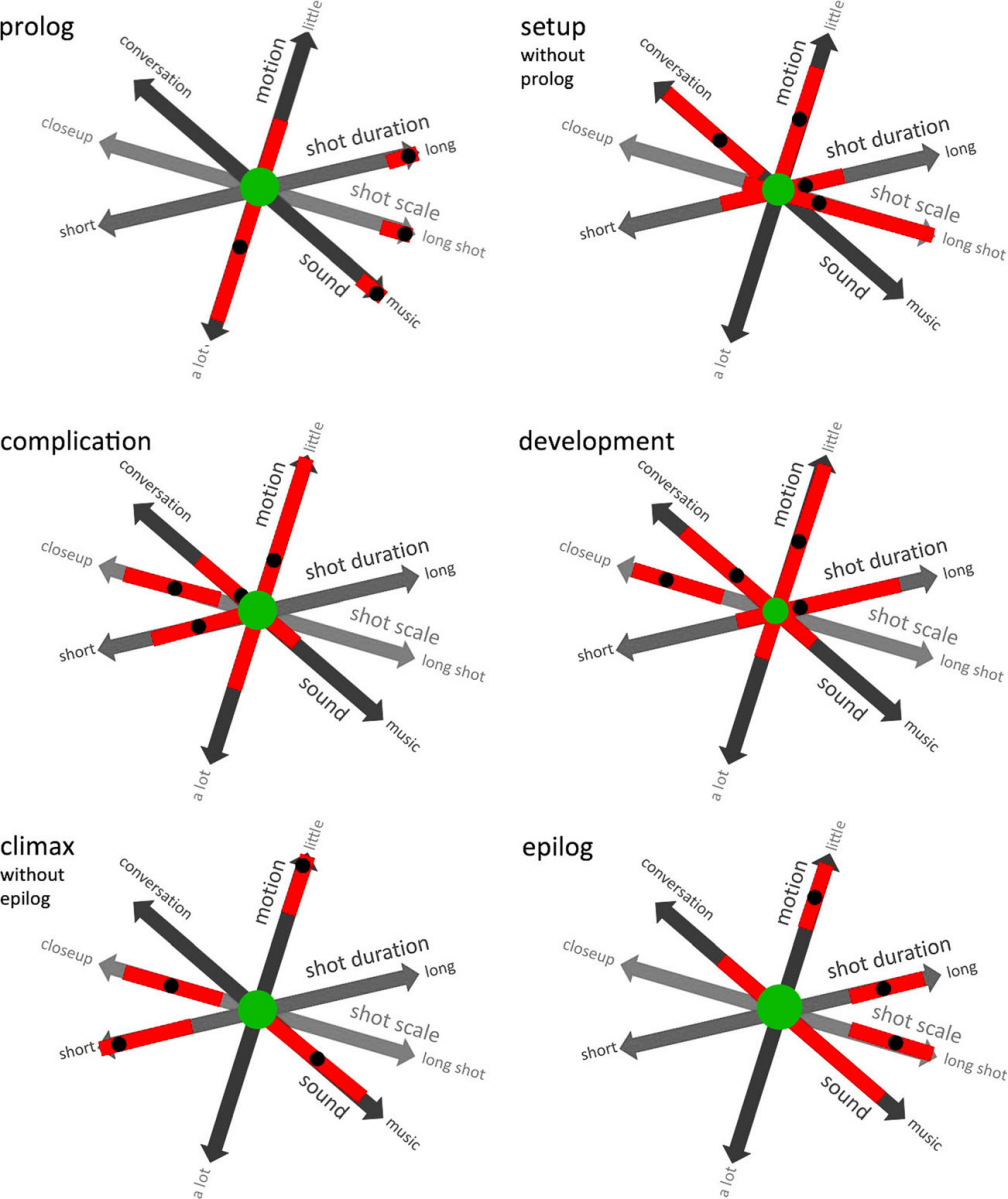
Distributions of values on five movie dimensions (as in Fig. 8) for six parts of movies—prolog, setup minus prolog, complication, development, climax minus epilog, and epilog. The extent of each bidirectional arrow corresponds to the 90th and 10th percentile of values in those domains, the black dot corresponds to the median value of each narrative part on that dimension, and the red bars correspond to the interquartile range. When black dots and red bars cover the gray arrow at the ends of the dimension, this means that those values extend beyond the 90th or 10th percentile. The size (area) of the green dots in the middle of each display corresponds to the luminance in the third principal component

Within the 100-bin framework, I consider Bins 1–3 to correspond to the prolog, Bins 4–25 to the setup minus the prolog, Bins 26–50 to the complication, Bins 51–75 to the development, Bins 76–97 to the climax minus the epilog, and Bins 98–100 to the epilog. Given that several of the five normalized dimensions of Fig. 8 were wildly skewed (shot scale from Study 7 and part of the purple dimension, and conversations from Study 8 and part of the green dimension), I created endpoint values of each dimension (at the arrow tips) to be equal to the 10th and 90th percentiles of those data. I then calculated the median value of each dimension (black dots) and the interquartile range (red bars) across each of the six parts of the narrative and fit these back onto the configuration in Fig. 8. The results are shown in the six panels of Fig. 9 as six glimpses of a trajectory, as Bordwell might have suggested in the epigram, through a narrational space. In addition, a filled green circle at the intersection of vectors in each panel represents the relative median amount of luminance in each section.

#### Results and discussion

The results show in a graphic manner the differences across the sequential parts of movie narration. The prolog has extreme values on three dimensions—a lot of nondiegetic music, long duration and long scaled shots—with varying motion and luminance. The values for the setup without the prolog generally converge to the center of the configuration except that the images darken only a bit and the nondiegetic music nearly drops out. The complication has a bit more music, and with shorter scaled and shorter duration shots and average brightness. Only motion remains roughly as it was in the setup, but even here it extends into the higher range. The development swings back on two of these dimensions—a bit less conversation, a bit longer shot durations, but also becomes darker, and keeping shot scales short while maintaining roughly the same amount of motion. The climax without the epilog adopts extreme values of elevated motion and short shot durations, maintains a short shot scale, has much more music and fewer conversations, and becomes markedly brighter. Finally, the epilog is bright, swings back markedly to very little motion and to long duration shots, but to a midrange of shot scales and a mix of conversation and music.

## Concluding discussion

### A theory of narrative structure in popular movies

Across the first 10 studies, I measured samples of films in 10 different ways accruing evidence from the syuzhets for a large-scale formula in the fabula of popular movies that has persisted for at least 70 years. A separate, smaller scale formula exists for the structure of scenes (Cutting, 2014; Cutting, Brunick, & Candan, 2012; Cutting & Iricinschi, 2015) and evidence for its independence was demonstrated in Studies 7 with shot scale. In addition, a larger scale whole-film set of patterns appears for nondiegetic music, and for shot scale in the smear of different scene onsets across many movies, as suggested in the results of Studies 5 and 10.

This medium-scale formula—actually a theory of popular movie narratives—is one that states that the fabulas of movies generally have four acts, as suggested by Thompson (1999) and Bordwell (2006), some with optional subdivisions within them. Basically, the formula is halfway between Aristotle and Horace—four acts of roughly equal duration, not three or five. These acts are the setup, the complication, the development, and the climax, with a likely prolog and epilog within the first and last acts, respectively. The fact that Studies 1, 2, and 4 find results that distinguish between bins in the complication and the development suggest that a three-act theory (Field, 2005) is insufficiently fine grained. The fact that no theory with more than four acts has specified any time frame on those acts makes them untestable in the manner that I have approached them. And the fact that the combined results in these studies are consistent with a theory proposing four acts with roughly equal durations suggests that it was amply tested. Let me review the evidence for this four-act structure and the likely psychological impact of those narrational measures on the viewer.

#### The prolog

The existence of a prolog within the setup is not part of any narrative theory of film that I know. It has been hiding, if not in plain sight, surely behind the ubiquitous opening credit sequence that has been used for more than 60 years. It is strikingly salient in almost every domain of data that I have investigated. That is, the first 5 to 7 min of narration (one, two, or three bins of the hundredfold divisions, or the first bin of the twentyfold divisions) are often markedly different than those that immediately follow. On average, shot durations are much longer (Study 1), noncuts more frequent (Study 2), nondiegetic music more prevalent (Study 5), shot scales longer (Study 6), more characters introduced (Study 7), fewer conversations heard (Study 8), and with more narrative shifts (Study 10) than anywhere else in the movie. Nonetheless despite its unique characteristics, like an epilog, not every movie has a prolog.

The existence of a prolog seems not to be an artifact of having opening credits superimposed on early shots. The pattern of shot durations in Study 1 is the same for those movies with and without overlaid credits; remember, the older movies with text-only title-card credits had these removed before the binning operation. Motion patterns are the same in both groups of films. The pattern of nondiegetic music also seems not related to the presence or absence of credits. My guess is that filmmakers introduced overlaid credits knowing that early shots of a films were already necessarily slow, and that placing credits over them solved the dual problem of familiarizing viewers with the locale and tenor of the story while also satisfying the need of putting the names of the major stakeholders up front. Many aspects of popular filmmaking are about optimizing resources, and overlaid credits with the normal content of the prolog allows a two-for-one fulfillment of needs.

It seems clear that the psychological import of the prolog is to win over the viewer and transport her into the narrative (Bruner, 1991; Cohen, Shavalian, & Rube, 2015; Gerrig, 1993; Green & Brock, 2000). It is the portion of the movie in which the filmmakers write their contract with the viewer. That contract states that what follows will be an absorbing and interesting story, and the viewers effectively sign on to be involved (Cutting, 2005). As Neil Brand suggested in the epigram to Study 5, viewers ready themselves “to experience some big emotions and . . . are on board for the ride.”

#### The setup

Thompson (1999), Field (2005), and others agree: the setup (including the prolog) introduces the characters and their issues. Study 7 provided strong evidence that this is the case. Indeed, 98% of all protagonists are introduced in the setup, and that those few who are not introduced are part of multiple protagonist teams. The setup also introduces 80% of all other important characters and the viewer gets to know them a bit. This rapid character introduction differs sharply from the first appearance of the various locations in movies, which are distributed more evenly through the narrative.

Beyond character introduction, the setup is separated from the complication by average shot duration (Study 1). By the end of the setup action shots, regardless of genre, may lift the physiological involvement of the viewer (Study 9). But basically, the viewer has dug into the narrative—with cognitive and perhaps a bit of physiological anticipation. She may have experienced the first plot point (the inciting action) and awaits the first turning point (the lock-in), trying to read the mind of the protagonist as to what she will do next.

#### The complication and the development

These acts are the domain of conversation (Study 8). The complication is markedly separate from the setup in character introduction (Study 7), scenes and subscenes begin to lengthen (Study 10), and the narrative begins to take off in a new direction after the protagonist has realized that her initial goals are thwarted.

The development also has several characteristics in contrast to the complication: its shot durations are a bit longer (Study 1), it has more noncut transitions (Study 2), and it is dimmer (Study 4) so that by its end the luminance falls to the psychological and literal “darkest moment” for the protagonist. Basically, it appears that these measures are indications that the story is getting more complex as it passes the midpoint.

#### The climax and the epilog

Evidence is strong for the climax as different from the development, and for the epilog as different from the rest of the climax. Conversation diminishes (Study 8), scenes and subscenes begin to shorten and narration time and narrative time converge (Study 10), shot durations plummet (Study 1), noncut transitions become fewer (Study 2), motion surges its highest levels (Study 3), brightness returns (Study 4), and nondiegetic music is at its most frequent since the prolog (Study 5). In viewers, heart rate and skin conductance should rise, and viewer absorption in the movie is likely to be at its peak. Many of these changes will last until the protagonist’s goal is attained, and then there is typically a turn into the epilog.

In the epilog shot durations then become longer than at any time since the prolog; dissolves and conversations may return; brightening continues. All is well, and cardiographic and electrodermal readings should begin to approach normal levels. The viewer can leave the theater much as she entered it, but having had an emotional ride and some intellectual exercise in theory of mind.

### Narrational dimensions and components

In the final two studies, through componential analysis, I condensed the variables of the 10 earlier studies to five dimensions based on film style. Filmmakers craft the syuzhet through decisions about staging, framing, editing, sound, focus, and color. I didn’t discuss focus or color here. The former can affect the relative amount of clutter in the shot (Cutting & Armstrong 2016), and the latter can be a powerful cue to scene and subscene change, particularly when flashbacks are involved (Cutting, Brunick, & Candan, 2012). However, I did investigate here the other aspects of film style: staging (Studies 3, 4, 7, and 8), framing (Study 6), editing (Studies 1, 2, 9, and 10), and sound (Studies 5 and 8). As it turned out, leaving luminance aside, rather than nine dimensions for these measurements of movies there are really only four, and even these are often strongly correlated.

One dimension concerns editing (shot durations, short vs. long and aligned with shot transition types, straight cuts vs. others), another concerns framing (shot scale, close-ups allied with the middle of scenes and known characters vs. long shots, the beginnings of scenes; and the entrances of new characters), a third concerns motion (fast vs. slow, with the former allied with action shots), and a fourth is about sound (with an opposition between conversations and nondiegetic music). Another dimension (lighting) is not strongly correlated with these four. Together, these five dimensions encompass central aspects of the basic toolkit available to filmmakers, who clearly make good and thorough use of it in crafting the structure of the narration to reflect larger narrative units. Values on these dimensions dance around considerably as the narration progresses, as shown in Fig. 9.

### What is the psychological import of these results?

One might accept these results but wonder what good they might be in psychological terms. First and most broadly, these results address an aspect of a decades-old issue in cognitive psychology; we talk about information processing but we often have no real idea how rich the information in the stimulus actually is; that is, what information is available to be processed. Although these studies measure no viewer responses, they do reveal variations in sources of information that have been shown to affect viewers.

This is important because movies and movie clips have been, and increasingly are, used as stimuli in psychological studies—of emotion (Hutcherson et al., 2005), of attention (T. Smith, 2013), of memory (Zacks, Speer, Vettel, & Jacoby, 2006), of cognition (Magliano et al., 1996), of aggression (Anderson, 1997), and of brain function (Hasson et al., 2008). I take this as a good sign, but if one is interested in understanding viewers’ responses, one should also be interested in all attributes of the stimulus. Consideration of some of the parameters explored here might contribute to a deeper understanding of viewers’ responses to movies. Moreover, psychologists are becoming increasingly interested in whole movies as a test bed for understanding perception, cognition, emotion, and other psychological responses more broadly (Kaufman et al., 2014; Seamon, 2015; Shimamura, 2013; Zacks, 2015), Clearly, movies are as complex, as interesting, but also different from real life.

Second, the normative data here provide strong evidence that the narration (the surface form of the narrative, or the syuzhet) typically contains many physical cues, continuously distributed, that likely guide attention and arousal and that could guide movie comprehension. That is, the “intricate web of character, event, time, and space” in the fabula becomes “transparently obvious” through the style conventions in the syuzhet (Thompson, 1999, p. 11). In the terms of McNamara and Magliano (2009, p. 301), these films are high in the ease of processing, and in this medium we viewers are high in our skill at extracting their information. This is important because, against some views of language structure, I would claim that aspects of form and meaning in popular movies are not independent. That is, there are strong correlations between the progression of the narration and the narrative states of movies.

Third, because psychological studies of text have focused on processing and comprehension, they have been confined to relatively short stories. Subjects can sit through only so much. To be sure, a great deal has been learned in this domain, but the structures of short stories are likely more impoverished than those of longer ones. Thus, the opportunity for understanding larger scale structures in textual narratives and how they might be processed may have been missed. Neither processing nor aspects of comprehension were studied here, but this analysis of movies allowed for the empirical explication of larger scale narrative structures in stories that may prove fruitful in work on discourse processes.

Finally, I would hope to reinvigorate the psychological study of multiple sources of information and preferably across dynamic situations, as suggested in Fig. 9. Most of our interactions in the world are surely guided by multiple information sources from which we select and combine cues. Examples of this kind of study can be found in Brunelli and Falavigna (1995); Christiansen, Allen, and Seidenberg (1998); Cutting and Vishton (1995), Harkins and Petty (1987); and Oruç, Maloney, and Landy (2003), but such investigations are not as common as one might hope for and almost nonexistent when coupled with the dimension of time.

### Narrative theory elsewhere

In the introduction I reviewed the proposed theoretical structure of plays and films. These were in terms of acts and turning points, and my analysis throughout this article has been fairly tightfisted in distinguishing among theories. Using Charles Darwin’s term for those who deal in taxonomies, I have been a splitter (F. Darwin, 1888, p. 105). Nonetheless, let me now swing the other way, become a lumper, and explore the similarities in narrative structure across the stories of different media.

There was considerable congruence across the different narrative theories of plays and movies: beginnings (exposition, setup, then inciting incidents and lock-ins) followed by middles (rising action and falling action, confrontation, complication then development) and then ends (climax then dénouement, twist, resolution, and/or epilog). To be sure, the exact ordering and segmentation of some of the parts shifted across different theoretical views. But more broadly there was great similarity, and that by itself is evidence for an art-form general and formulaic narrative structure.

Of course plays and movies are not the only narrative forms following this kind of scheme. Labov and Waletzky (1967) analyzed short oral histories and life narratives given by inner-city adults and gleaned a structure that included six sections. The first, the *abstract*, is perhaps like a prolog—optional, short, and declaring what the story is about. The *orientation*, similar to a setup and some exposition, is followed by the *complicating action* (their term), in which something goes awry. This section carries the bulk of the story. Often, next is an optional *evaluation,* in which the speaker steps out of the narration to tell of its import (a feature rare in movies, but see *The Big Short,* 2015), as if to keep the listener attentive; then there is the *resolution*, or climax, which describes the outcome of the conflict; and finally a *coda,* like an epilog, which marks the close. Moreover, it should be stressed that these oral histories are unrehearsed and spontaneously offered. Thus, people with little formal education can spontaneously generate this kind of narrative structure. In turn, this suggests a deep well of shared cultural knowledge about how stories are told. Adding generality, Longacre (1976) presented a nearly identical scheme for oral stories across cultures.

In a different vein, there is a traditional Asian four-part story structure *(kishōtenketsu*) found in Japan, Korea, and China (see, e.g., Berndt, 2013). This has strongly influenced manga, the comic books that developed in Japan after World War II (Gravett, 2004). Okabayashi (2007), for example, describes these parts. Part 1 is the *ki*, where the story begins and the characters are introduced and begin to interact. The opening frame typically provides an establishing shot on the location in which the story will take place, much like that in a movie prolog. Part 2 is the *shō*, where suspense is built up and the tempo gradually increased as the characters confront conflicts and try to achieve their goal. Okabayashi calls this the development stage although it seems to combine with aspects of the complication or confrontation in movies. Part 3 is the *ten*, a dramatic and unexpected turn of events. This seems to have the sense of a combination of the climax with a twist. Finally, Part 4 is the *ketsu* or conclusion. It resolves the conflict, but unlike a traditional epilog, it may leave some loose ends for a subsequent adventure in next installment of the story.

Again in the medium of visual narratives, Cohn (2013, 2015) outlined the structure of visual sequential narratives and matched five parts to other forms of storytelling. The first part is the *establisher*, which sets up an interaction but without any action. This seems analogous to a setup. This part is followed by an *initial*, which initiates the tension in the narrative to follow, which seems analogous to an inciting incident or to the complication. Third is the *prolongation*, which continues the trajectory of the protagonist’s path, which seems analogous to the development. Fourth is the *peak*, which marks the height of the narrative tension. Pretty clearly, this is a climax. And finally there is the *release*, which dissolves the tension of the interaction. Again, this seems very close to one of the functions of an epilog.

Consider again literature, where Watts (1996) in his self-help book on writing a novel proposed a story arc of eight points: the *stasis* (like the prolog, setup, or exposition), the *trigger* (like the inciting incident), the *quest* (like the lock-in on a path toward achieving the goal), the *surprise* (one or a series of derailing events like those found in the complication), the *critical choice* (like the transition at the end of the complication into the development where the protagonist doubles down to achieve the goal), the *climax*, the *reversal* (as in the epilog, where the new normal is established, often with characters’ roles reversed), and the *resolution* (where loose ends of the plot are tied up, also as in the epilog).

And Vladimir Propp (1928/1968), in his analysis of Russian folk tales, proposed a more articulated but similar structure. Within the 31 elements of stories that he elaborated, the folktales themselves all generally begin with an introduction (like a prolog), and then within a *first sphere* someone goes missing, a hero is warned of portending events, or a villain seeks information or engages in some trickery, all of which could conform to a setup with an inciting incident. This is then followed by a *second sphere*, which contains the body of the story. Here, the hero is challenged in some way or reaches some destination to face evil. This has many attributes of a complication. The *third sphere* may contain the donor sequence, where the hero receives something from possibly a magical agent, that allows him or her to accomplish his goal often against a villain, and then achieves that goal. This has the characteristics of a development with a climax. Finally, there is the *fourth sphere*, or the hero’s return, where there can be some additional complications (twists) where he or she is unrecognized by the townsfolk, or there is a false hero, but then finally gets accepted—essentially, as in a dénouement or epilog.

Finally, even a previous psychological analysis of stories has adopted a theoretical framework that has considerable overlap with all of these other schemes. Mandler’s (1978) story grammar, for example, has a *beginning* (like a prolog or setup), a *reaction* (like an inciting incident or complication), an *attempt* (goal-seeking behavior like that found in the confrontation, or across the complication and development), an *outcome* (like the resolution or climax), and the *ending* (like the dénouement or epilog). And see van Dijk (1977) for a similar framework with five parts—setting, complication, resolution, evaluation, and moral, which is a bit like four acts and an epilog.

In other words, narrative formulae are similar across movies, plays, oral histories, manga, comic strips, novels, and folktales. To be sure, these have been honed within given domains, all the while keeping the overall story form. Movies are no different. They have a narrative structure with a chain of events that link causes and effects, that involve one or more protagonists, who have desires and seek a goal, have the path to that goal blocked in some way, and then try to overcome it, and if achieving that goal bring back, or establish a new, local social order. These parts have film-style correlates in shot durations and transitions, motion, luminance, scale, contrasts between conversations and nondiegetic music, and patterns of character introduction and scene changes—all cues in the syuzhet for the viewer to construct the fabula*.*

Finally, these story-form analyses—from movies in this article, elsewhere in manga, folktales, and the rest—are all schema based. That is, they emphasize patterned norms of story production, of narration. Psychological analyses of story comprehension used to take a schema form (e.g., Brewer & Lichtenstein, 1981; Mandler & Johnson 1977), but such approaches tend to rely heavily on memory. The field of narrative comprehension over the last 30 years has moved on to consider more active contributions of the listener/reader/spectator with organizing concepts like spreading activation, attentional focus, constraint satisfaction, and inference (McNamara & Magliano, 2009). This is not to say schemas are not important for comprehension, but they are not currently regarded as a central focus.

My view is that for the moviegoer the patterned results reported here help trigger dynamic changes in physiological states—motion and emotion, music and emotion, cuts and attention, luminance and affect polarity, and more. But these results also reflect schemas. Schemas allow for more rapid processing of the story, and we know that speeded processing is correlated with engagement and positive affect (Pronin, 2013). A full understanding of movies is not yet on the horizon, but I hope these results can help formulate a path to take us there.

### Epilog

One may well ask, as does J. Hillis Miller (1990, p. 68), “*why do we need stories at all?”* (italics in the original). His answer is pertinent and uncontroversial: As children or adults we learn from stories; stories are the backbone of education; they can reinforce and even create cultural values and promote system justification (Jost, Banaji, & Nosek, 2004), but they can also subvert those values in ways that vary from the relatively innocuous to beyond the edge of insolence (Žantofský, 2014). Given the striking sameness of narrative structure in the varied media outlined above, Miller also asks a second question: “*Why do we need the ‘same’ story over and over?”* (italics in the original). He continues (1990, p. 70):

The answers to this question are more related to the affirmative, culture-making function of narrative than in its critical or subversive function. If we need stories to make sense of our experience, we need the same stories over and over to reinforce that sense making. Such repetition perhaps reassures by the reencounter with the form that the narrative gives to life. Or perhaps the repetition of a rhythmic pattern is intrinsically pleasurable, whatever that pattern is.

There is no question that popular movies give pleasure to a great many members of our culture, and none that artistic repetitions in culture also give pleasure (see Cutting, 2006). Moreover, on the basis of the data presented here, there should be no question that popular movies are patterned, and patterned in the same general way, so that such repetitions could be part of that pleasure making.

Movies are among the most deeply entrenched and widely appreciated art forms of contemporary life. Indeed, the British Film Institute (2012) reported that the average person in the United Kingdom claims to have seen 87 movies per year across all media platforms. Surely the viewing habits in the United States and those in other Western countries are similar. Such viewing frequency is astonishing and, I would claim, tells us that the narrative form of popular movies feeds into and fits deeply within our minds. In psychology, we deal so often (and successfully) with near-tachistoscopic moments—a word, a list, a sentence, an isolated decision, a glancing expression, and a fleeting movement. But our daily experience and our interactions with many arts occur over a larger time frame and are full of structure and meaning. Indeed, the kind of larger scale periodic structure found in movies may reveal a lot about what our minds like best and the rhythms they prefer.

## Footnotes

1 How movies are made, of course, involves millions of dollars, thousands of people, and some of the most advanced digital technology in the world—none of which is relevant in this context. I am interested in the general surface form of movies, not how they got that way. There is also a debate in the humanities literature about which comes first, the *fabula* or the *syuzhet* (Culler, 1981, pp. 170–172; Derrida, 1979). My view is that there are two fabulas, one that precedes the syuzhet (in the storyteller) and one that follows it (in the story consumer).

2 Note that *catastrophe* (Greek for “down twist”) didn’t inherently mean death and destruction for the protagonist, but since the terms *drama* and *tragedy* were essentially interchangeable, it has come mean so.

3 It is difficult to compare the “lengths” of text and movies, but calculations for a typical movie script are one page for every minute of screen time. Scripts are rather spare—double-spaced, wide margins, and lots of dialog—but even at only 100 words per page, these stories are as much as 12,000 words or more without considering the visual information that provides additional narrative exposition.

4 Some differences between English-language movies and those from non–English-speaking countries are addressed in Cutting and Candan (2015).

5 I would prefer to call these *narrational shifts*, but given the precedent in the literature, I will stay with narrative shifts.

6 Barry Salt initially suggested this technique in discussions about the results of Cutting, Brunick, and DeLong (2011a) to correct an error there. It resulted in a second paper (Cutting, Brunick, & DeLong, 2012) using a finer-grained technique than used in Study 1 and yielding a less striking result.

7 There is no need to adjust the *R* ^2^ in the test data because the fits are parameter free.

8 Imagine the incongruity of a dissolve into a shot of a T. Rex chomping into a jeep.

9 Previously, my students and I created what we called a visual activity index (VAI; Cutting, DeLong, & Brunick 2011a), which is one minus the correlation value. This made all increasing values correspond to more motion, but it also seemed a bit cumbersome, so I simply use correlation values here. In that research we also correlated every other frame (Frames 1 & 3, 2 & 4, etc.) rather than adjacent frames as I have done here (Frames 1 & 2, 2 & 3, etc.). The difference is trivial.

10 The downsampling was done to reduce computation time, and the choice of a square frame neutralized effects of different aspect ratios (horizontal/vertical extends of the frame). Aspect ratios among the larger sample of films were 1.37, 1.5, 1.66, 1.75, 1.87, 2.2, 2.35, and 2.55.

11 When testing for statistical significance among data where at least one of the pair of correlated values represents 20 original bins rather than 100, the degrees of freedom represent the lower *n*.
